# Probing mechanical interaction of immune receptors and cytoskeleton by membrane nanotube extraction

**DOI:** 10.1038/s41598-023-42599-9

**Published:** 2023-09-20

**Authors:** Fabio Manca, Gautier Eich, Omar N’Dao, Lucie Normand, Kheya Sengupta, Laurent Limozin, Pierre-Henri Puech

**Affiliations:** 1 CNRS, INSERM, Laboratoire Adhesion et Inflammation (LAI), Aix Marseille University, 13009 Marseille, France; 2 CNRS, Centre Interdisciplinaire de Nanoscience de Marseille (CINaM), Aix Marseille University, 13009 Marseille, France; 3 Turing Center for Living Systems (CENTURI), 13009 Marseille, France

**Keywords:** Biological physics, Biophysics, Immunology, Lymphocytes

## Abstract

The role of force application in immune cell recognition is now well established, the force being transmitted between the actin cytoskeleton to the anchoring ligands through receptors such as integrins. In this chain, the mechanics of the cytoskeleton to receptor link, though clearly crucial, remains poorly understood. To probe this link, we combine mechanical extraction of membrane tubes from T cells using optical tweezers, and fitting of the resulting force curves with a viscoelastic model taking into account the cell and relevant molecules. We solicit this link using four different antibodies against various membrane bound receptors: antiCD3 to target the T Cell Receptor (TCR) complex, antiCD45 for the long sugar CD45, and two clones of antiCD11 targeting open or closed conformation of LFA1 integrins. Upon disruption of the cytoskeleton, the stiffness of the link changes for two of the receptors, exposing the existence of a receptor to cytoskeleton link—namely TCR-complex and open LFA1, and does not change for the other two where a weaker link was expected. Our integrated approach allows us to probe, for the first time, the mechanics of the intracellular receptor–cytoskeleton link in immune cells.

## Introduction

The importance of mechanics and mechanotransduction, at both molecular and cellular scales, is now well recognized in cell biology in general^1^ and in immunology in particular^2^. In the context of immunology, T cells, and the T cell receptors (TCRs), have a special significance in being the very first players in adaptive immunity. Mechanics of T cells has been studied using a variety of techniques^3^, recently revealing that T cells have atypical mechanical responses^4,5^. Likewise, mechanics of the interaction of the TCR and its molecular partner, the peptide loaded Major Histocompatibility Complex (pMHC), is a subject of current research with some groups reporting a catch bond^6,7^, and some others not^8^. A key to understanding how molecular scale mechanics and chemical kinetics are translated to cell scale mechanical behavior may be the bio-chemical link between intracellular moiety of molecular linkers and the cell cytoskeleton^9,10^. The identity of the chain of proteins that form this link, often forming a molecular clutch, is well-known from experiments on non-immune cells, and for adhesion molecules like integrins where a hierarchy of actin-binding proteins like talin and vinculin, among others, are recruited to clusters of bound integrins^11^; however, the nature of this link is still elusive for TCR where it has been called a condensate^12^, perhaps to emphasize the physical, rather than chemical, nature of the interactions.

Cytoskeletal reorganizations are essential for correct functioning of leukocytes, including response after activation^2,9,13–16^. Like in other cell types, leukocytes, including T cells, exert forces mainly through their actin cytoskeleton. Forces are generated as a result of actin polymerization/branching and myosin-induced contractions. The details of rearrangement of the actin meshwork during adhesion and spreading was reported for T cells^17,18^. The polymerization of actin at the cell edge leads to spreading^19,20^ and to actin retrograde flow close to the cell interface, that drags newly formed clusters of TCR towards the center of the spreading cell^21^. This drag force, of frictional origin, to which all membrane receptors linked to the interfacial actin cytoskeleton—including both TCR and integrins—are exposed, is transmited through the linkers to the underlying substrate^4,20,21^, which in turn has been shown to lead to sustained signaling^22^.

While the cross-talk of the cytoskeleton with signaling is well documented for T cells^23,24^, the details of the signaling cascade associated with mechanotransduction has been reported in only a few studies^4,25–27^. It was shown that T cells can be activated simply by force application on TCR alone^13^, via a Src kinase-dependent process^28^. It is thus clear that force is an important control parameter of molecular function (especially in leukocytes). Interestingly, unlike in most other cell types, the sensing of mechanical environment in T cells appears to be myosin independent^4,20^; the extent of its spreading, when mediated by TCR alone, is biphasic with substrate stiffness^4,5^. T cells spread increasingly better on stiffer substrate, but only up to a point, after which the harder the substrate, the lesser the spreading^4,5,29,30^. Such a behavior can be a result of the TCR-ligand bond being a catch bond, as modelled in the context of early spreading of fibroblasts^31^, but it could also be explained by a model that considers the mechanics and kinetics of the entire molecular assembly that links the cytoskeleton to the substrate^4^.

**Figure 1:**
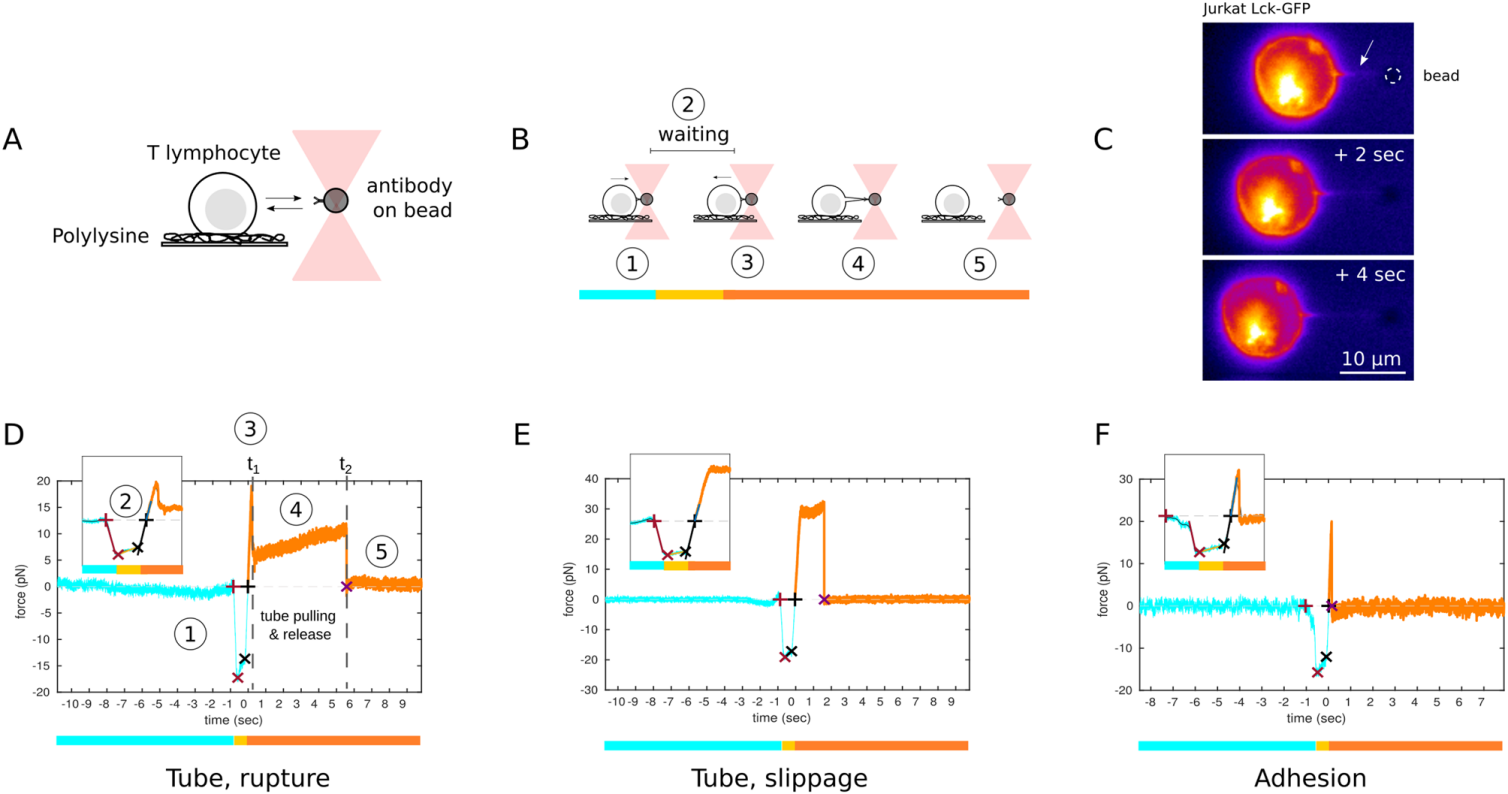
Principle of tube extraction using optical tweezers and representative force curves. (**A**) A trapped colloidal bead coated with antibodies interacts with the surface of an adhered T cell. (**B**) The cell is brought in contact with the bead (blue stripe) $\textcircled {1}$, followed by a waiting period at constant position (yellow stripe) $\textcircled {2}$, and then pulled back (orange stripe) $\textcircled {3}$. This can lead to the extraction of a membrane tube $\textcircled {4}$, which eventually breaks $\textcircled {5}$. (**C**) Fluorescence micrographs of the process of tube pulling from a membrane labelled T cell. (**D**–**F**) Typical force vs. time curves (insets are presenting zooms over the contact region). (**D**) Tube with discontinuous jump, called ‘rupture’ (numbers correspond to stages shown in **B**). The time at transition from $\textcircled {3}$ to $\textcircled {4}$ is $t_1$ and from $\textcircled {4}$ to $\textcircled {5}$ is $t_2$. (**E**) Tube with a discontinuity and no jump, called ‘slippage’. (**F**) Binding and unbinding without tube formation, called ‘adhesion’.

The role of the membrane-to-cortex attachment in regulating cell protrusions was recently emphasized for formation of cell protrusions in general^32^. In the context of integrin mediated adhesion, they can stabilize robust cell adhesion under flow^33^, and mediate leukocyte rolling^34^. Similar elongated membrane structure like microvilli play an essential role in the exploration of its environment by a T cell^35,36^, via TCR molecules located to the tip of the structure^37^. In all these examples, the link between receptors and cytoskeleton is difficult to characterize mechanically due to accessibility issues.

The application of a force localized to the membrane achieved eg. by using an antibody as a molecular handle allows to test the links to the extracellular part of a specific membrane-bound receptor^38,39^. Pulling on these links, for example to break a ligand/receptor bond, may eventually link to extruding thin membrane tubes, and is one of the popular methods to probe membrane tension and mechanics^40^.

Tubes can be extracted from pure membrane systems such as giant unilamellar vesicles (GUV)^41,42^ or even artificial membranes^43^. Theoretically, the extrusion of nanotubes has been studied via analytical models^44^ and Monte Carlo simulations^45^. These models allow to link the force-extension curve of the tube’s extrusion to the mechanical properties of the membrane. Even if a small force overshoot can be seen, the experimental force vs. time curves of GUVs tube pulling are essentially monotonous^44,46^.

Moreover, tubes can also be pulled from living cells^47,48^, in order to probe viscoelasticity of the cell^49,50^, and can be complicated by the presence of the membrane to cytoskeleton links under force^38,51–53^. Such experiments are usually described theoretically via models that take into account the viscoelasticity of the cell^44,54–57^, including in the context of de-adhesion from the cytoskeleton^38,46,56^. The theoretical analysis is complicated by the need to take into account the presence of membrane-to-cortex attachment (MCA) molecules^58^. The (few) existing theoretical studies are almost limited to numerical analysis^53^, which allow a comparison with extrusion experiments but do not permits a direct fit of the extrusion curve. Of note, experimental curves very often show a “peak then plateau” shape^52,59,60^, and only the plateau force is used to estimate the membrane tension and/or an attachment energy to the cytoskeleton, not providing any details of a molecular mechanism between the probed protein(s) and actin, but rather global membrane/cortex attachment^52,61^. The precise significance of this peak, that is a reminder of the breaking of a bond, has been rarely adressed both experimentally and theoretically^46^.

An integrated mechanical model including effects of membrane, actin cortex and specific receptors is so far missing. In the present work, we propose a contribution under the form of an analytical model that allows to fit the force-elongation curves of nanotube extrusion, and considers explicitely the presence of the force peak upon retraction, before a plateau-like regime. We also describe in the same model the case where this peak is absent. Although the model does not give access to the underlying molecular mechanisms of the extrusion (e.g. phase transition of the phospholipids), it permits to extract separately the effective contributions of the elasticity provided by the membrane and the molecules. We demonstrate its efficiency by studying the case of the proteins composing the immune synapse, probed at the membrane of a living T cell. These proteins were predicted to exhibit differential interaction strengths with the actin, allowing the apparition of complex, biphasic, spreading behavior on activating substrates^4^. The link of these proteins to the actin cortex represents an essential mechanism linking molecular structures, such as the TCR and the adhesion molecules, to the mechanosensitive elements that participate actively in the early T cell activation^15^.

**Figure 2:**
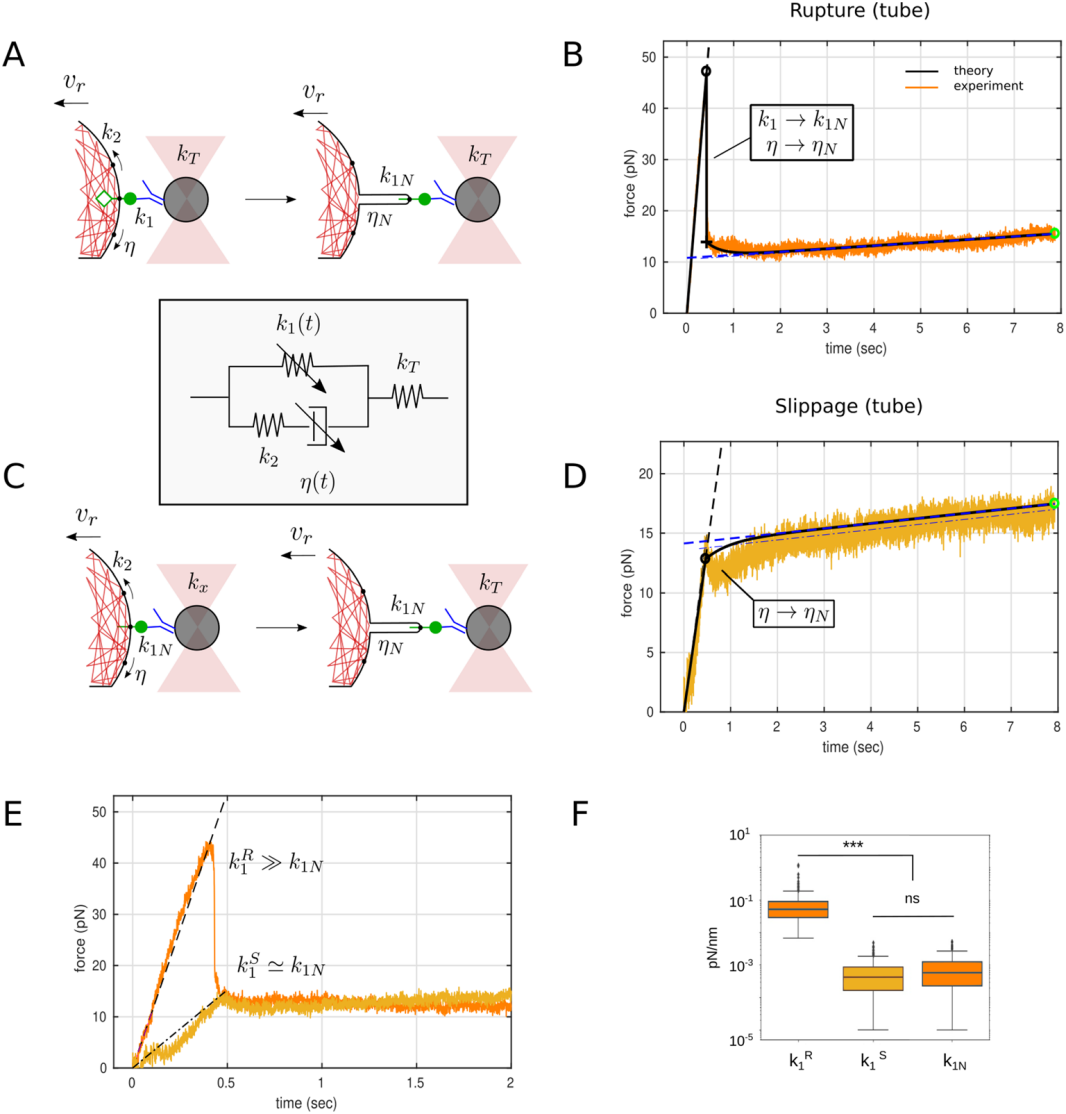
Microscopic interpretation of force curves and mechanical model (in inset). (**A**) In the presence of a receptor–cytoskeleton link (hollow green diamond), its rupture leads to a tube formation, with corresponding changes in viscoelastic parameters $k_1 \rightarrow k_{1N}$ and $\eta \rightarrow \eta _N$ (see variable elements $k_1(t)$ and $\eta (t)$ with an arrow in Inset). (**B**) Corresponding ‘rupture’ force curve showing a discontinuous jump upon rupture. (**C**) In the absence of a receptor–cytoskeleton link, a tube is pulled with membrane “slippage” on the cytoskeleton, implying $k_1 = k_{1N}$ and a transition $\eta \rightarrow \eta _N$ only. (**D**) Corresponding ‘slippage’ force curve showing a simple discontinuity and no jump. In (**B**,**D**), the black line corresponds to the fit to the data. (**E**) Overlay of typical rupture and slippage force curves to illustrate that, after the rupture event at $t_1=0.5$ s, the two curves are similar. (**F**) Measurements of $k_1^{(R)}$ and $k_{1N}$ for rupture curves (N = 116 for each) and $k_1^{(S)}$ for slippage curves (N = 165) (Inset). Viscoelastic model consisting of a spring $k_1$ representing the stiffness of the receptor-to-cytoskeleton link, in parallel with a series consisting of a second spring $k_2$ and a dash-pot with viscosity $\eta$ representing the effective rigidity and viscosity of the cell cortex. A spring $k_T$, in series with the whole, accounts for the stiffness of the optical trap. Note that $k_1(t)$ and $\eta (t)$ are time dependant piece-wise functions that encompass the mesoscale transitions leading to the formation of a membrane tube.

Here we access the mechanics of the putative link between the main lymphocyte membrane receptors, among them the TCR, and the actin cytoskeleton by pulling membrane nano-tubes from T cells, using antibody-coated beads in an optical trap. The time evolution of the force is fitted using a viscoelastic model that consists of springs representing either molecular or cellular elasticity and dash-pots that take into account the cellular and tube viscosity. By analysis data using scenarios corresponding to cases where the membrane receptor detaches or not from the cytoskeleton during tube formation, we are able to separate cellular and molecular elasticities. Finally, we compared hundreds of curves from experiments using different antibodies as molecular handles to access various membrane bound receptors.

## Results and discussion

### Experimental system

To dissect the interaction between immune receptors and actin cytoskeleton, we used optical tweezers to pull membrane tubes from Jurkat T cells. The cells, non activated and gently adhered onto polylysine glass substrates, were used to contact, for short duration ($\le$ 1 s) and weak pushing forces ($\le$ 20 pN), beads decorated with antibodies directed specifically against a given molecule (Fig. 1A,B), eventually leading to a small fraction of the events ($\le$ 30 %) corresponding to the pulling of membrane tubes (Fig. 1C, similar to earlier reports^52^) and leading to force vs. time curves of specific morphologies (Fig. 1D–F). To exploit the richness of these curves, we developped a mechanical model encompassing molecular and cellular scales, together with the dynamics of the tube pulling (Fig. 2, inset, see below).

**Figure 3:**
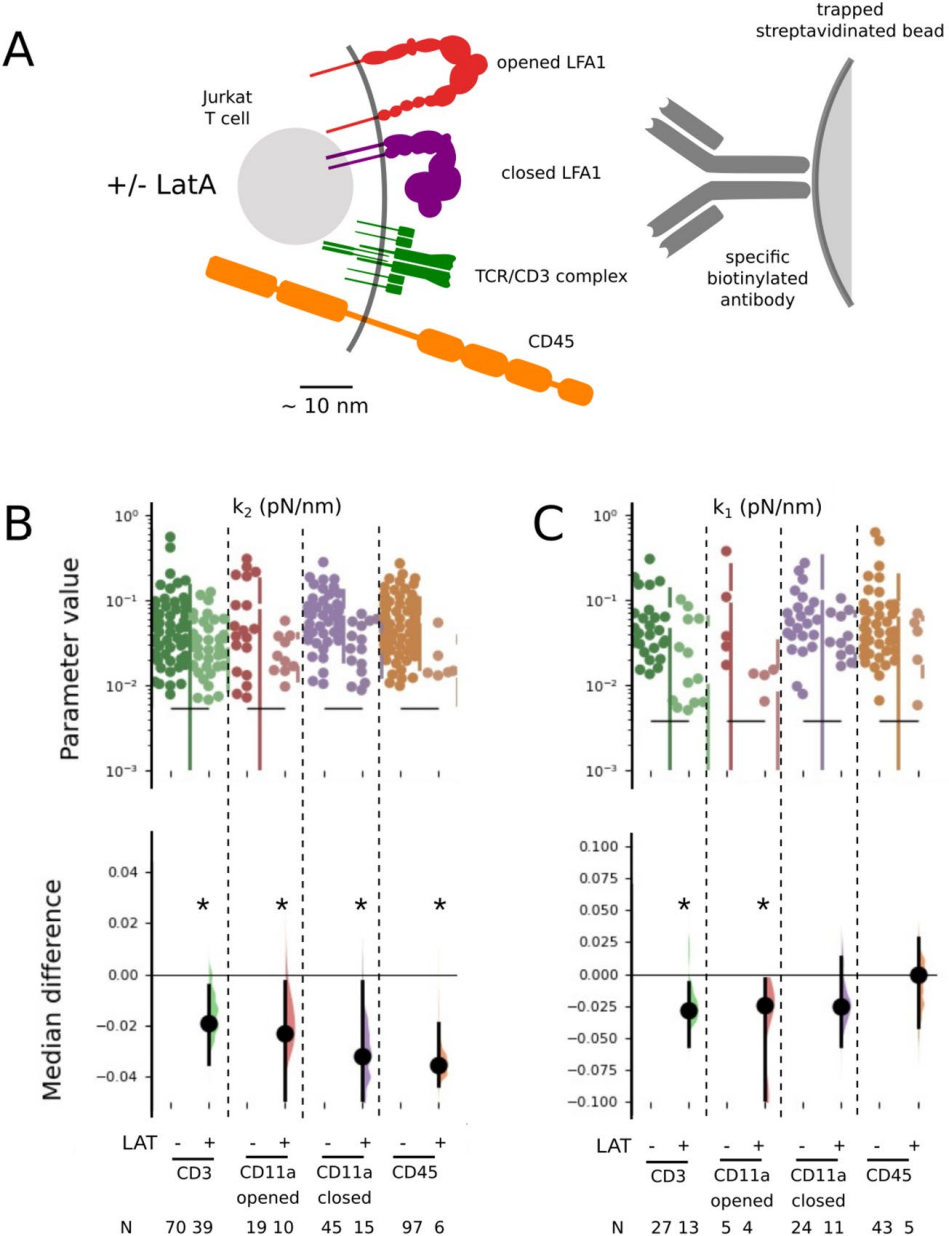
Effect of cytoskeleton perturbation on elastic parameters measured for each membrane receptor targeted on the T-cell membrane. (**A**) Schematic of the receptors and their conformations which were specifically solicited by appropriate antibodies on the bead used to pull membrane tubes. (**B**) Scatter plot of $k_2$ (effective cell cortex stiffness) (upper graph) and median difference plot with estimation (lower graph) to compare, for each antibody, the cases +/− Latrunculin. (**C**) Same as (**B**), for $k_1$ (stiffness of receptor–cytoskeleton link). N indicates the number of curves for each case. Star (*) indicates a significant difference of medians ($p<0.05$) following Dabest analysis (see SI).

To interrogate some of the essential transmembrane proteins involved in T cell activation^4,62^, and also in IS formation, we used four molecular handles under the form of antibodies, to target the TCR/CD3 complex, the integrin LFA1 in its closed or open conformations and the long CD45 molecule (Fig. 3A). As positive and negative controls for the interaction with the cytoskeleton, we used that opened LFA1 is known to have a stronger interaction with actin than its closed or intermediate conformation^62^. To our knowledge, the situation is largely unknown for the TCR/CD3 complex^4,62^, and no clear data exists for CD45^63^. To destabilize the actin cytoskeleton, hence perturbating its possible links to the probed molecules, cells were challenged with a low concentration of Latrunculin A (hereafter LatA).

### Force curves morphologies and transitions

Visual inspection of roughly 8900 curves revealed four morphologies. First, and most interesting, about 4% of the curves exhibit a clear spike-like discontinuity followed by a slow increase and a second discontinuity where the antibody–receptor bond breaks and the force goes to zero, henceforth called “rupture” case Fig. 1D). Second, 6% of the curves show a step-like discontinuity followed by slow increase and a step down to zero force, henceforth called “slippage” (Fig. 1E). Third, 23% exhibit a spike which immediately falls to zero force, called “adhesion” (Fig. 1F). As expected, due to short and gentle contact parameters imposed in order to fulfill single molecule conditions, a fourth case is seen in the vast majority (67%) of the curves, where no attachment of the bead to the cell occurs, and no meaningful force-curve is obtained (not shown here). Of note, the slowly rising plateau seen in the first two cases is characteristic of tube extraction^38,52,56^. Of note, the interaction leading to the tube pulling or adhesion events between the bead and the cell is specific and linked to the relative abundance and accessibility of the different surface molecules targeted (Fig. S1).

We interpret the difference between the two tube cases in molecular terms. In the rupture case, the spike/discontinuity corresponds to the rupture of the cytoskeleton–receptor link and a concomitant tube formation, which were not experimentally separable in time (Fig. 2A,B). In the slippage case, the receptor-to-cytoskeleton link is either absent or very weak, and the membrane slips over the actin cortex and a tube forms without having to rupture any specific linkage (Fig. 2C,D). Finally, the force abruptly falling to zero, seen in the adhesion case (Fig. 1F, Fig. S2, and eventually at late times for tubes, corresponds to the breaking of the extracellular antibody–receptor bond, leading to the detachment of the bead from the receptor handle. In some cases, the tube was not rupturing at the end of the experiment, due to a finite total pulling length hence duration, leading to “infinite” tubes. All these cases can be interpreted in the frame of our mechanical model.

### Mechanical model

The relevant part of the experimental system and its equivalent mechanical model are pictured in Fig. 2, inset. The mechanical model is essentially a standard linear solid model^54,56^ representing the cell-tube-receptor system, in series with another spring to account for the optical trap. The former consists of a spring with spring constant $k_1$ that represents the stiffness of the receptor-to-cytoskeleton link as well as the tube that is to be pulled, in parallel with a second spring, $k_2$, and a dash-pot, with viscosity $\eta$ , representing the effective rigidity and viscosity of the cell. An additional spring $k_T$, in series with the whole, accounts for the stiffness of the optical trap. It is important to include $k_T$ as it was previously shown that neglecting the stiffness of the handle—here the OT—may lead to significant over or underestimation of the mechanical properties of molecules^64,65^.

Importantly, in our model, $k_1$ and $\eta$ are not constant over time, and drop, at particular moments of tube pulling, as $k_1 \rightarrow k_{1N}$ and/or $\eta \rightarrow \eta _N$, following different scenarios that we explicit hereafter.

At time $t=t_1$, the receptor-to-cytoskeleton link ruptures and the membrane detaches from the cytoskeleton leading to the formation of the tube. The stiffness of the link ($k_1$) is not expected to be time dependent while it is intact, and similarly, the stiffness of the tube ($k_{1N}$) is considered to be time independent. The cell elasticity ($k_2$) is not expected to be impacted by tube pulling, however, the viscosity, with potentially major contribution from the membrane itself, may change (from $\eta$ to $\eta _N$). Thus, $k_2$ is constant and $k_1(t)$ and $\eta (t)$ are piece-wise constant. $k_T$ is experimentally set and constant while the tube exists. At the end, the tube detaches due to deadhesion of the receptor–ligand bond, $k_T$ then (effectively) goes to zero and the force falls to the baseline value. This sequence is clearly reflected in the force curves (see Fig. 1D,E).

The constitutive model of the coupled system is then given by the following differential equation

1 $$\begin{aligned} \frac{df(t)}{dt} \alpha (t) + f (t) \beta (t) = \frac{dx(t)}{dt} \bigl [ k_1(t)+k_2 \bigr ] + x(t) \left[ \frac{dk_1(t)}{dt}+\frac{k_1(t) k_2}{\eta (t)}\right] \end{aligned}$$

where $\displaystyle { \alpha (t) = 1 + \frac{k_1(t)+k_2}{k_T}}$, $\displaystyle \beta (t) = \frac{k_2}{\eta (t) } + \frac{1}{k_T}\frac{dk_1(t)}{dt} + \frac{k_1(t) k_2}{\eta (t) k_T}$. The imposed distance as a function of time is given by $x(t) = v_r(t)\times H(t)$, where *H*(*t*) is a Heaviside function, $v_r$ (the pulling speed) is imposed at time $t=0$ (which corresponds to $f=0$ when starting to pull on the system, Fig. 2).

The response is evaluated by solving the differential constitutive equation separately before and after the discontinuity at $t=t_1$. The analytical solution, and its comparison with the numerical solution, can be found in SI (see SI Eq. 2). This solution is a general case of the classical standard-linear-solid model (SLSM)^54,56^, with an additional spring $k_T$, and where time discontinuities are introduced for both $k_1(t)$ and $\eta (t)$. The solution at $t\le t_1$ deviates from a linear behavior expected from purely elastic contributions ($k_1+k_2$), and describes the relaxation caused by the viscosity of the cell, $\eta (t)$. The solution at $t>t_1$ describe the relaxation of the system after the rupture of the link ($k_1 \rightarrow k_{1N}$), and the concomitant transformation of the locally flat cell membrane into a tube ($\eta \rightarrow \eta _{N}$), which results in a plateau-like shape in the force evolution (Figs. 1D,E, 2B,D). SI Eq. (2) was used to fit all the experimental curves in order to obtain the value of the mechanical parameters.

The pipeline for fitting the curves is detailed in SI. The rounded median values of the fixed and fitted parameters, pooling data from all conditions, are given in Table 1. While $k_1$ is explicitly determined here for the first time to our knowledge, the obtained values of other mechanical constants are overall coherent with literature^38,56^. Explicitly, Ref.^38^ reported a value equivalent to $k_1+k_2 = 0.3$ pN/nm which compares well with our value of 0.1 pN/nm for $k_1$ and $k_2$; Ref.^56^ reported values equivalent to $k_2= 0.2$ pN/nm (0.05 pN/nm here) and $k_{1N} = 0.001$ pN/nm (0.0005 pN/nm here).

**Table 1:** Physical parameters of the model (median values on the entire data set).

Parameter	Symbol	Value	Units	R	S	A
Discontinuity time	$t_1$	0.25	s	✓	✓	✗
Molec. stiffness	$k_1$	0.05	pN nm$^{-1}$	✓	$k_{1N}$	✓
Tube stiffness	$k_{1N}$	0.0005	pN nm$^{-1}$	✓	✓	✗
Cell stiffness	$k_2$	0.05	pN nm$^{-1}$	✓	✓	✓
Cell viscosity	$\eta$	0.04	pN nm$^{-1}\,\hbox {s}$	✓	✓	✓
Tube viscosity	$\eta _N$	0.008	pN nm$^{-1}\,\hbox {s}$	✓	✓	✗
Pulling velocity	$v_r$	2500	nm$^{-1}$	–	–	–
Trap stiffness	$k_T$	0.25	pN nm$^{-1}$	–	–	–

*R* rupture, tube, *S* slippage, tube, *A* adhesion, no tube, *tick/cross symbol* parameter accessed or not by the model (resp.).

### Mechanical transitions observed between the different tube morphologies are coherent

On one hand, as prescribed by our fitting to the model, $k_1 > k_{1N}$ in the “rupture” case. On the other hand, we observe that the values for $k_{1N}$ are similar for the “rupture” or “slippage” cases for tubes (Fig. 2E,F, Fig. S3A,B) , corresponding to the fact that the situations become similar when the intracellular bond is broken and $k_1$ reaching $k_{1N}$ (“rupture”) and when starting from it (“slippage”). Interestingly, $k_1$ is similar for adhesion events and “rupture” tubes (Fig. S3A), while $k_2$ is not dependent on the event being a tube or an adhesion (Fig. S3C). Moreover, the viscosity $\eta _N$, ie. after all transition(s), is the same for the two cases with a tube, corresponding to a similar tube pulling mechanism. Importantly, all of these observations are independent from the precise molecular handle that was used to pull the tubes, showing the consistency of our model and methodology. Interestingly, one can appreciate that $k_{1N}$ is not affected by LatA, while $\eta$ seems to be decreased in all cases, together with $\eta _N$ (Fig. S3).

We present the distribution of times $t_1$ and $t_2$ on Fig. S4A,B, respectively. $t_1$ corresponds to the time of the cell to (cell + tube) transition. In the rupture case, it is the simultaneous transition of $k_1$ and $\eta$, while for slippage case, it is the transition of $\eta$ alone. Figure S4C shows no difference of $t_1$ between rupture and slippage cases. This validates our approximation that the two transitions are detected simultaneously for the rupture case in our experiments. Note that the lifetime of the tube is $t_2-t_1 \sim t_2$ since $t_2 \gg t_1$. Then, since the pulling velocity $v_r$ is constant, the length of the tube is proportional to $t_2$. We observed that the tube length can be larger than cell size and is limited by the maximal pulling distance of the optical trap set-up ($\le 20\, \upmu$m).

### Immune receptor interactions with cytoskeleton are molecule specific

Figure S5A,B present the cellular elasticity, $k_2$, and the molecular bond parameter, $k_1$, which correspond to the intracellular bond of the handle to the cytoskeleton, respectively. The results obtained for $k_{1N}$, $\eta$ and $\eta _N$ can be found on Fig. S6. None of the five parameter appears to be affected by the particular handle used, which allows to conclude that the molecular details of the *extracellular* interaction between bead and cell are not affecting our measurements.

Notably, when perturbating the cytoskeleton with low doses of LatA, the drug affected the global cell mechanics, as expected, which can be seen on the homogeneous and significant effect on $k_2$ values, decreasing them (Fig. 3B). Remarkably, LatA did not affect the intracellular molecular bond parameter $k_1$ the same way for the different handles (Fig. 3C). While a strong and significant effect is seen for the opened conformation of LFA1, no significant effect can be seen for the closed conformation, even if the median shift is similar, in agreement with the relative interactions of the two conformations with actin. Interestingly, the case of CD45 was not showing any sensitivity to the drug. For TCR/CD3, we observed a significant effect of the drug. Taken together, we see a differential effect of the drug on $k_1$ that we interpret as a differential interaction of the proteins with the cytoskeleton.

Let us now explore in greater detail the possible meaning of $k_1$, that, by its very nature bridges the molecular scale (a few nm), and the mesoscopic tube scale (100 nm).

To do so, $k_1$ is further decomposed into a circuit of springs as shown in Fig. S7, such that $k_{1} = \frac{k_{\perp }\times k_{\parallel }}{k_{\perp }+ k_{\parallel }}+k_{1N}$. From Table 1, we already know that $k_{1N} \ll \frac{k_{\perp }\times k_{\parallel }}{k_{\perp }+ k_{\parallel }}$. We will further require that, in an unperturbed state, $k_{\parallel } < k_{\perp }$: a statement that we will substantiate below.

Physically, we identify $k_{\perp }$ as the elasticity of the direct link between the intracellular part of the surface-molecule that is bound to the antibody handle and which transmits a force locally roughly perpendicular to the membrane and parallel to its own mechanical axis. $k_{\perp }$ is a local property of the solicited receptor and is therefore expected to be antibody dependent. $k_{\parallel }$ represents the elasticity associated with the weaker links of other membrane bound receptors which are necessarily pulled along when the membrane is pulled, a mechanism somewhat similar to the frictional breaking proposed by Groves^66^ or stick-slip mechanisms mooted in the context of mechanosensing^4^, with the force being transmitted parallel to the membrane. $k_{\parallel }$ is a non-local, mesoscopic parameter, which is not specific to the receptor that is bound and therefore it is expected to be antibody independent. $k_{1N}$ can be thought of as the elasticity associated with the emerging tube when it comes into being, and probably represents residual non-specific interaction between the bulky intracellular moieties of membrane-bound receptors and the intracellular environment, making it the softest spring in the circuit.

This relatively simple mechanical circuit captures the behaviour of all the antibodies tested under force and in presence/absence of latrunculin. Let us consider each case separately.

- *The unperturbed system*: for all four antibodies, namely aCD3, aCD11a-open, aCD11a-closed and aCD45, the shape of the circuit ensures that the response is dominated by the softer spring in series—namely $k_{\parallel }$. $k_{\perp }$, which is expected to be the antibody dependent element but also the stiffest element in the circuit, is not probed. Naturally, whatever the antibody, the response is identical, dominated by the non-specific $k_{\parallel }$ component, measured here to be 0.05 pN/nm. The only parameter reported in literature that is akin to $k_{\perp }$ was for CD3, in the context of cell spreading and mechanotransduction^4^, where it was reported to be 0.3 pN/nm—an order of magnitude stiffer than $k_{\parallel }$ reported here—consistent with our hypothesis.
- *Perturbation of the actin cytoskeleton*: perturbing actin using latrunculin is expected to strongly impact the direct link between the target receptor and the actin cytoskeleton. With a large dose of latrunculin, we can expect all existing links to be severed. However, at the small dose of latrunculin used here, we are left with many cases of rupture, indicating that in these cases, the link survives and it is these cases where we measure $k_1$. Latrunculin, at low doses, should not impact $k_{\parallel }$, which is expected to depend on the meso-scale connectivity of the actin network.

In all cases, at high force or extension, the link, presumably $k_{\perp }$, ruptures and the response is then dominated by $k_{1N}$.

It is important to note that the attribution of each element to specific molecular players is, given the state of art, necessarily speculative but the basic mechanical arguments based on the spring network is strictly validated by our experimental data.

### Model exploration and predictions

**Figure 4:**
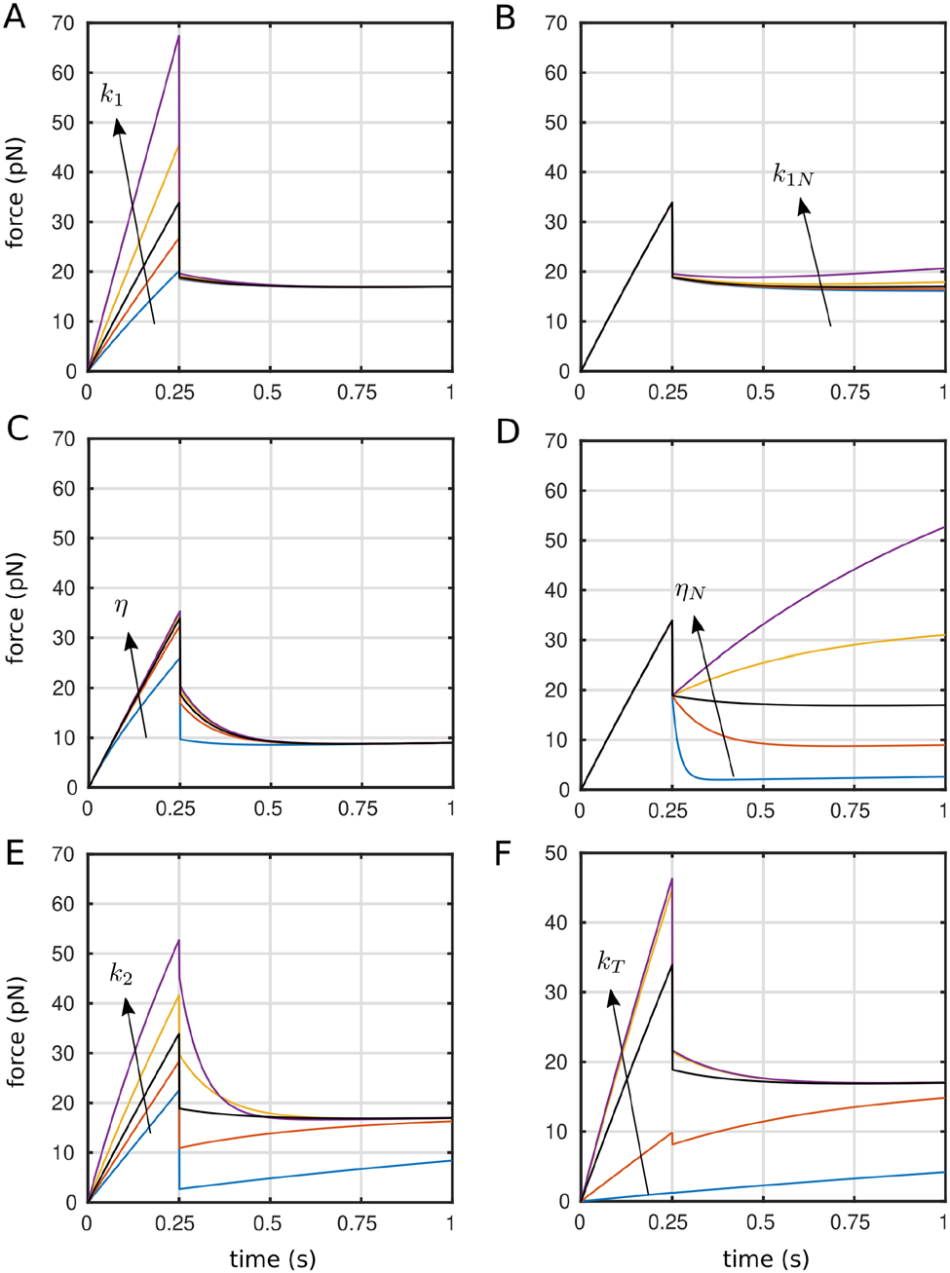
Parametric study of the viscoelastic model for the rupture case. Reference model parameters are obtained from the fit of the experimental ‘rupture’ curve in Fig. 2B: $v_r=2000\,\hbox {nm}\,\hbox {s}^{-1}$, $k_T=0.25\, \hbox {pN}\,\hbox {nm}^{-1}$, $k_1=0.05\, \hbox {pN}\,\hbox {nm}^{-1}$, $k_2=0.05\, \hbox {pN}\,\hbox {nm}^{-1}$, $\eta = 0.04\, \hbox {pN}\,\hbox {nm}^{-1}\,\hbox {s}$, $k_{1N}=0.0005\, \hbox {pN}\,\hbox {nm}^{-1}$, $\eta _N = 0.008\, \hbox {pN}\,\hbox {nm}^{-1}\,\hbox {s}$, and $t_1 = 0.25\,\hbox {s}$. For panels (**A**) to (**E**), the corresponding reference curve is shown in black while the other curves have been obtained by multiplying by 0.1, 0.5, 1, 2 and 5 the reference parameter value: (**A**) $k_1$; (**B**) $k_{1N}$; (**C**) $\eta$; (**D**) $\eta _N$; (**E**) $k_2$. For panel (**F**), $k_T$ = 0.01, 0.1, 1, 10 or 100 $\hbox {pN}\,\hbox {nm}^{-1}$ and the other parameters have their reference value.

To assess the robustness of parameter determination, we performed a parametric study of the model (Fig. 4 for “rupture”; Fig. S8 for “slippage”), to dissect the effects of variations of the different fitting and fixed parameters. As expected, the early-time quasi-linear behavior is mainly governed by $k_1$, which does not affect the post-rupture part of the curve (Fig. 4A). To the contrary, the value of $k_{1N}$ affects only the residual slope of the force for $t>t_1$ (Fig. 4B). Coherently with our observations made when fitting the data, variations in tube viscosity $\eta$ has minimal impact before $t_1$, and only a moderate one after, (Fig. 4C). $\eta _N$ governs the trend of the force from a convex to a concave behavior for $t>t_1$ (Fig. 4D). Aside, the shape of the relaxation (concave or convex) depends on the value of $t_1$ (Fig. S9). Interestingly, large variations of $k_2$ have only a small impact on the linear loading phase, but $k_2$ however plays a crucial role for $t>t_1$ (Fig.  4E), and controls for the slippage case the maximal force at $t_1$ and curvature after it (Fig. S8). Notably, the behavior of the force-curve also depends on the stiffness of the force transducer, and we scan the typical range of common force-spectroscopy measurements, going from photon-field (softer) to mechanical (stiffer) transducers, showing the profound impact of the measuring spring on the morphology of the force vs. time data curve (Fig. 4F)^67^ .

We can observe that $k_{1N}$ and $\eta$, when varied over orders of magnitude do not greatly modify the curves, all other parameters staying constant to values relevant to the ones measured in this report. This is reflected in the observed large distributions of the values of these parameters when extracted from the data (Fig. S6A). Interestingly, $\eta _N$ appears to have a large impact on the curve shape, which is indeed reflected in a more compact distribution of this parameter. Such a better and more sensitive determination of $\eta _N$ also reveals that this parameter is, in almost all cases significantly, sensitive to LatA, which decreases its value (Fig. S6B). This particular dependence may need a closer examination of the tube structure, in particular its actin content if any, to link it to the biology.

In order to better test our model, the examination of either the same samples with different techniques or different samples with optical tweezers may be relevant as proposed below.

First, pulling tubes using springs of different rigidities using the same antibodies and cells would allow to test the effects of $k_T$. Changing the bead size, material and the power of the laser could allow to vary $k_T$ within 0.2–1 pN/nm. Atomic force microscope single molecule force mode could be used to ramp up $k_T$ from typically 10 pN/nm to several 100 pN/nm^60^. For rigidities below 0.2 pN/nm techniques based on glass fibers could be used.

Second, working with (1) the same receptor, with a constant density at cell surface, but with (2) a tunable strength of interaction with the cytoskeleton would allow to better test our model. Possible targets could be integrins (such as LFA1 here) since one can tune their extension, activity and binding to the cytoskeleton using ions in solution or the state of T cells, active or not^62^. Antibodies directed toward low, medium and high affinity integrins, which are collapsed to extended in shape and weakly to stronly bound to the cytoskeleton, renders this feaseable. Another interesting possibility would be to harness the role of ERM family proteins to play on membrane linkages (see $k_\parallel$), by eg. using KOs or coupling optogenetic local stimulations and membrane tube pulling. Aside, tuning the polymerization/depolymerisation state or dynamics of the actin could allow to separate the effects of given compounds or mutations on $k_1$ and $k_2$ for receptors of interest, as revealed here for LatA and TCR.

Overall, we explored a wide range for the values of the parameters, and conclude that the model’s predictions—both qualitative and quantitative—are robust. Most importantly, the model is highly sensitive to $k_1$, which is the principle parameter of interest in the present study.

## Conclusions

Our data, model, and the fitting presented here demonstrate that pulling of a tube may (rupture case), or may not (slippage case), involve breaking of an internal bond, distinct from the external antibody/antigen bond. The signature of this breaking is contained in the force curve (Fig. 1) where an increase followed by an abrupt fall signifies “rupture” whereas a gentle increase and smooth fall signifies “slippage”. This interpretation is supported by previous work of Nowak et al. who showed that pulling of cytoskeleton-associated membrane tubes involves higher force and a more abrupt jump as compare to pulling pure membrane tubes^46^. Concentrating on the former (rupture) case, the internal rupture involves snapping of the spring $k_1$ in Fig. 1, which upon rupture takes on its residual value of $k_{1N}$. We have further shown that the value of $k_1$ is latrunculin dependent for some immune synapse proteins, in particular for the TCR complex. Our quantitative analysis of the cellular and molecular parameters of our model is then coherent with our precedent modeling of T cell bi-modal spreading on substrates of variable elasticities^4^.

Overall, we can conclude that the intracellular molecular spring linking the receptor to the cytoskeleton, with stiffness $k_1$, originates from two main components that are not explicitly introduced in our 1D model of dynamical tube pulling, in addition to its residual value ($k_{1N}$) associated with the tube after bond rupture. These two main components are: $k_{\perp }$ which is a spring-like element perpendicular to the local membrane plane and collinear to the traction force. It corresponds to the actual molecular interaction with the actin, and $k_{\parallel }$, an element parallel to the membrane, encompassing the interaction of the cytoskeleton with the rest of the membrane including proteins which spans it. The first element is drug dependent and we propose that the second, which corresponds to an intermediate scale is essentially independent of the exact details of actin to receptor interaction (Fig. S7).

The interpretation presented above show that we do probe differential interactions of IS proteins with the actin cytoskeleton by using different antibodies as molecular handles, but the difference cannot be revealed without perturbing the system using a drug. The stiffness response of the molecular spring corresponding to the specific link to actin, $k_{\perp }$ is “hidden” due to the presence of a softer spring in the system. We can only state that for all receptors targeted here, the value of $k_{\perp }$ in an unperturbed cell is larger than 0.05 pN/nm. To dissect the exact values or origin of $k_{\perp }$, more refined experiments, such as using cells with specific KOs of ERM molecules, talin or other putative adapter molecules needs to be performed, with large enough data-sets to reveal potentially subtle differences in the fitted parameters. Nevertheless, here we demonstrated the importance of the mesoscale, represented by the membrane and its association with the actin network, for a full understanding of the IS at the molecular scale. A proper understanding of this intermediate scale may be key to how immune cells convert molecular cues to cell scale activation.

## Material and methods

Details about the experimental, analytical and numerical procedures can be found in the supplementary materials section.

### Supplementary Information

Supplementary Information.

## Data Availability

The datasets used and analyzed during the current study available from the corresponding authors on reasonable request.
