# Supplementary material for: Probing mechanical interaction of immune receptors and cytoskeleton by membrane nanotube extraction
Source: Sci Rep. 2023 Sep 20;13:15652. doi: 10.1038/s41598-023-42599-9 (PMC10511455; doi:10.1038/s41598-023-42599-9)
Supplement: Supplementary file 1 — Supplementary Information. [file 41598_2023_42599_MOESM1_ESM.pdf]

Supplementary Material

Probing mechanical interaction of immune receptors and cytoskeleton by membrane nanotube extraction. Manca et al.

Content

- Material and Methods
- Supplementary Figures
- SI References

Materials and methods

Cell culture

Jurkat E6-1 cells (ATCC, #TIB-152) were grown at 37°C and 5% CO_2 in Roswell Park Memorial Institute (RPMI) 1640 1x medium (Gibco, #11875) supplemented with 10% fetal bovine serum (FBS) and 1% stabilized L-glutamine (GlutaMAX, Gibco). They were diluted in fresh medium every 2 or 3 days in order to keep the concentration between 0.4 10^6 cells/mL and 1.2 10^6 cells/mL.

Beads preparation

We used polystyrene beads of diameter 2 μm pre-coated with streptavidin (Polysciences, Inc., #24160), having an initial concentration of 3 10^9 beads/mL. They were diluted to 1/10 in Dulbecco's Phosphate-Buffered Saline 1x (DPBS) with 1% (w/v) Bovine Serum Albumin (BSA, Sigma Aldricht) to reach a volume of 250 μL. They were washed three times by centrifuging during 6 min at 6,700 g and replacing the supernatant with 250 μL of DPBS/BSA 1%.

After the last wash, beads were resuspended in 100 μL of DPBS/BSA 1% and a solution containing 10 μL of biotinylated antibody at 0.5 mg/mL was added. We used biotinylated mouse IgG2aK monoclonal antibodies (all from eBioscience, Thermofisher, Biorad): anti-CD45RO (clone UCHL1), anti-CD3 (clone OKT3, clone UCHT1), anti-LFA1 (closed conformation, anti-CD11a clone 38 ; opened conformation anti-CD11a clone HI111). We used as an isotype control a non specific IgG2aK (clone eBM2a). Beads and antibodies were co-incubated 30 min at room temperature (RT) under stirring. Beads were then washed as previously and finally resuspended in 500 μL DPBS/BSA 1%, to a final concentration of $\sim 10^8$ beads / mL. The functionnalized beads were stored during maximum one month at 4°C.

Sample preparation

Petri dishes having 35 mm diameter and 0.17 mm thick glass bottom (Fluorodish, WPI, #FD35-100) were incubated 30 min at RT with 2 mL of polylysine solution (Sigma-Aldrich, #P8920) diluted to 1/10 and washed three times with 2 mL DPBS 1x.

Approximatively 5 10^5 cells were taken from the culture one day after splitting, and resuspended in pure RPMI after gentle centrifugation during 3 min at 400 g; they were then transferred to the Petri dish. They were incubated 30 min in culture conditions to allow them to adhere. Medium was then gently replaced by supplemented RPMI and 10 μL of beads solution corresponding to 1-1.5 10^6 beads just before installing the sample on the heating microscope stage.

To perturb actin, latrunculin A (Sigma-Aldricht) was used at a low final concentration of 2 to 5 μM, incubated 30 min with cells before the experiment. Note that the experiments were performed in the presence of the drug. We verified experimentally that the presence of a small residual amount of DMSO in the experiment medium did not affect the measurements.

Optical tweezers

The acquisition of force curves was performed with a Nanotracker 2 (JPK Instruments/Bruker) optical trapping device, equipped with a motorized/piezo stage, mounted on an inverted microscope (Axio Observer, Zeiss). The sample was fixed on a thermoregulated petridish holder (PetriDish Heater, JPK Instruments/Bruker), the temperature of which was set to 37°C for all the experiments.

The trapping objective (C-Apochromat 63x/1.2 W Corr, Zeiss) was covered by a drop of immersion oil (Immersol W 2010, Zeiss) that has a refractive index near to the one of water (n=1.334 at 23°C) . The detection objective (W-Plan-Apochromat 63x/1.2 W Corr, Zeiss) was immersed in the sample medium. The optical trapping laser had a wavelength of 1064 nm and a maximal power of 3 W. The laser was focused in the medium by the trapping objective and the out-coming beam was driven through the detection objective to quadrant photodiodes. These allow to measure the displacement of the trapped object in the back focal plane in three dimensions and to quantify the forces after calibration.

For transmission light microscopy, a LED lamp is focused on the sample by the detection objective and the picture is acquired by a CCD camera (DFK 31BF03.H, Imaging Source).

The NanoTracker software (version 6+ on GNU/linux, JPK Instruments/Bruker) controls the position of the objectives, the position of the sample, the position of the trap, the intensity of the laser and the attenuation filters before the detection photodiode.

The distribution of bead diameters was measured separately on bright field microscopy images and the average value was used in all experiments ($2R \simeq 1.67 \pm 0.07$ μm). We imposed a medium viscosity η of 6.96 10^{-3} Pa/sec. The stiffness of the trap is calibrated by the software based on the spectral analysis of the thermal noise implemented in the control software (1).

A ramp designer allows to program the motion of the sample with the piezoelectric stage. The ramps had three phases: first, a rectilinear motion toward the cell interrupted when the force detected by the photodiode exceeds a given threshold (10 or 15 pN); then, a pause of a given duration in which the sample stays immobile (0 to 1 sec) and the force relaxes; and finally, a rectilinear motion in the opposite direction until a given distance is reached (15-20 μm). The speed of the forward and backward motions was typically set at either 2 or 2.5 μm/sec. The acquisition frequency for the force curve data was 2048 Hz.

During the experiment, the force signal in three dimensions, based on the stiffness calibration along the three motion axis, is recorded and saved. In order to optimize the force to be colinear to the relative motion of the bead and cell, we attempted to have the trajectory perpendicular to the cell membrane. For this, to minimize lateral forces, , when the trajectory was in X (resp. Y) axis we incrementally adjusted the Y (resp. X) and Z positions to minimize the force measured in Y (resp. X) and Z axis before the first cell / bead contact.

The measurements, which are saved as compressed and encoded commercial format files, were finally converted by using the NanoTracker data processing software (JPK/Bruker) into tab separated text files that can be feeded into our Matlab procedures.

Model

The general solution of Eq. 1 (see main text) is given by

$$f_{tot}(t) = f_a(t)[1 - H(t - t_d)] + f_b(t)H(t - t_d). \quad (2)$$

with

$$f_a(t) = f_0 e^{-\frac{k_2\gamma_1}{\eta}(t-t_r)} + \left\{ \frac{x_0\, k_T}{k_1+k_T}\left[k_1 + \frac{k_T k_2}{k_1+k_2+k_T} e^{-\frac{k_2\gamma_1}{\eta}(t-t_r)}\right] + \frac{v_r\, k_T}{k_1+k_T}\left[k_1(t-t_r) + \frac{k_T\eta}{k_1+k_T}\left(1-e^{-\frac{k_2\gamma_1}{\eta}(t-t_r)}\right)\right]\right\} H(t-t_r), \tag{3}$$

$$f_b(t) = \frac{k_T}{\left(k_{1N}+k_T\right)^2}\left\{\left[\left(k_{1N}^2 + k_{1N}k_T\right)\left(x_0 + v_r(t-t_r)\right) + k_T\eta_N v_r\right] + \frac{e^{-\frac{k_2\gamma_{1N}}{\eta_N}(t-t_d)}}{\left(k_{1N}+k_T\right)^2}\left[f_d^+\left(k_{1N}+k_T\right)^2 - k_T^2\eta_N v_r\left(k_{1N}^2 k_T + k_{1N}k_T^2\right)\left(x_0 + v_r(t_d-t_r)\right)\right]\right\} H(t-t_r), \tag{4}$$

where, we considered $k_1(t)$ and $\eta(t)$ as piecewise functions

$$k_1(t), \eta(t) = \begin{cases} k_1, \eta & t \le t_d, \\ k_{1N}, \eta_N & t > t_d, \end{cases} \tag{5}$$

being t_d the time at which the discontinuity happens, t_r the retraction time ie. the time at which the retraction starts, x_0 the initial position of the optical bead, f_0 the initial force measured by the tweezers, v_r the speed of pulling and $\gamma_1 = \frac{k_1+k_T}{k_1+k_2+k_T}$, $\gamma_{1N} = \frac{k_{1N}+k_T}{k_{1N}+k_2+k_T}$.

The value of the force after the discontinuity is equal to

$$f_d^+ = k_x[x(t_d) - x_T(t_d^+)] = k_x[x(t_d) - x_T(t_d^-)\Gamma],$$

being $x(t)$ the total length of the system, $x_T(t)$ the distance of the optical trap from its equilibrium position and $\Gamma = \frac{k_1+k_2+k_T}{k_{1N}+k_2+k_T}$ the ratio of the effective stiffnesses in x_T before and after t_d.

Finally, getting the recorded position of the optical bead from Eq. 2, $x_T(t_d^-) = x(t_d) - f_a(t_d^-)/k_T$, and being $x(t_d) = v_r \times (t_d - t_r)$, we have

$$f_d = k_x[v_r(t_d-t_r)(1-\Gamma)] + f_a(t_d^-)\Gamma.$$

Notice that, with $t_d = t_1$, and explicitating the force $f_a(t_d^-) = f_a(t_1^-)$, we get the explicit solution of $f_b(t)$ (see Eq.7), which is a function of the free parameters $k_1, k_{1N}, k_2, \eta, \eta_N, t_1$ only.

From Eq.7, with the boundary conditions we choose to offset the raw data to, which are $f_0 = 0$, $x_0 = 0$ and $t_r = 0$, we obtain the following simplified form of the time-force evolution

$$f_a(t) = \frac{k_T v_r}{(k_1+k_T)^2}\left[k_T\eta\left(1-e^{-\frac{k_2\gamma_1}{\eta}t}\right) + k_1(k_1+k_T)t\right], \tag{6}$$

$$f_b(t) = \frac{k_T v_r}{(k_{1N}+k_T)^2}\left[k_T\eta_N\left(1-e^{-\frac{k_2\gamma_{1N}}{\eta_N}(t-t_1)}\right) + k_{1N}(k_{1N}+k_T)(t-t_1)\right] + \frac{k_T v_r e^{-\frac{k_2\gamma_{1N}}{\eta_N}(t-t_1)}}{(k_{1N}+k_T)^2(k_{1N}+k_2+k_T)}\left\{k_1^2(k_{1N}+k_2)t_1 + k_1k_T\left[(2k_{1N}+k_2)t_1 + \eta\right] - \eta k_T(k_1+k_2+k_T)e^{-\frac{k_2\gamma_1}{\eta}t_1} + k_T\left[k_{1N}k_T t_1 + \eta(k_2+k_T)\right]\right\}, \tag{7}$$

Notice that, in Eq.6, for $k_T \to +\infty$, $k_1/k_T \to 0$, we recover the classical solution of the standard-linear-solid model (SLSM) (2,3). With Eq. 2, we fitted all the experimental curves and obtained the distribution of the parameters.

In Fig. S10, we report the theoretical results, both numerical and analytical, of the force evolution $f_{tot}(t)$ obtained for the input $x(t)$. The system parameters have been fixed to values similar to the ones reported in Table 1: $k_T = 0.25\text{pN}\,\text{nm}^{-1}$, $k_1 = k_2 = 0.05\text{pN}\,\text{nm}^{-1}$, $\eta = 0.02\text{pN}\,\text{nm}^{-1}\,\text{s}$, $k_{1N} = 0.0005\text{pN}\,\text{nm}^{-1}$, $\eta_N = 0.004\text{pN}\,\text{nm}^{-1}\,\text{s}$, and $t_1 = 0.25\text{s}$. The red curve corresponds to the numerical resolution of Eq. 1 (with a Runge Kutta 4th order integrator) and the dotted-black curve corresponds to the analytical solution of the force of Eq. 2. For reasons of numerical stability, the Heaviside functions $H(t)$ have been replaced by a step-like function defined as : $\mathscr{H}(t-t_j) = \frac{1}{2}\big\{1 + \tanh[\alpha(t-t_j)]\big\}$, with $\alpha = 10^7$.

Data Analysis

The analysis of the force measurements consisted of three major steps. First, the classification of the curves, which automatically identifies their characteristics. Second, the fitting of the curves, which gives the estimation of the mechanical parameters. Third, the statistical analysis of fitted parameters given by the first two steps.

Steps one and two have been done in an automatic fashion *via* an *ad-hoc* MATLAB code named “u-Tubes”. (see Algorithm below). The first part of the code is devoted to the treatment of the data, including : smoothing of the signal, baseline correction, characteristics points detection, optical artifact detection and correction. For each curve, several observables are measured and stored, such as the slopes around zero force at contact and release, the slope of the tube (if any), the relaxation during the contact phase, and many others (≃140 in total). These measures were exploited for the direct estimation of the physical parameters and some of them served as guessing parameters for the fitting procedure. The most important task of this first part is the classification of each curve in the three main categories - “contact”, “adhesion”, “tube” (either finite or “infinite”) - along with, for the tubes, the classification of the two type of discontinuities “rupture” and “slippage”. The second part of the code, settles the fitting of the data by means of the proper model related to adhesion, ”rupture” tube or ”slippage” tube. The outcome of this part are the fitted parameters (t_1, t_2, k_1, k_2, η, k_{1N}, η_N, see Table 1) and a supplementary classification of the curves on their fitting “quality” based on the examination of fit convergence and magnitude of residuals.

Finally, the parameter values obtained in the two first steps were prepared for statistical analysis and representation using a set of Python scripts (with the use of Python `dabest` supplementary package, https://acclab.github.io/DABEST-python-docs/index.html, (4)). The details of each of these parts are presented below.

μ-Tubes algorithm

1: Read data (txt file from JPK)
2: Smooth data (moving average)
3: **procedure** Classification
4:     Detect characteristic points (contact, wait, retraction)
5:     Detect & Correct optical artefact
6:     Measure geometrical parameters (forces, slopes, etc.)
7:     Classify discontinuity : rupture, slippage
8:     Classify curve : contact, adhesion, finite/infinite tube
9:     Estimate mechanical parameters (k_1^{est}, k_2^{est}, etc.)
10: **end procedure**
11: **procedure** Fitting
12:     Assign model and constraints (depending on 8. & 9.)
13:     Determine guessing values (k_1^{guess}, k_2^{guess}, etc.)
14:     Fit curve and get parameters (k_1, k_2, etc.)

Evaluate fitting quality from residuals
end procedure
Save and Plot data

Curves processing

Raw curves were first smoothed with the built-in MATLAB function `smooth` (with a moving average algorithm). The next step of the data treatment was the spotting of characteristic time-force points coordinates.

Characteristic points

These points mark a discontinuity in the force curve and delineate the boundaries for the curve segmentation in the three consecutive parts: contact, wait, retraction. In Fig. S11 (corresponding the panel A of Fig.2), from left to right, t_{c1} and t_{c2} are the time points at which the contact starts and ends respectively (in red); t_{r1} and t_{r2} are the start/end retraction points (in black); t_{d1}^- and t_{d1}^+ are the first discontinuity in the force curve during retraction (left and right limits), t_{d2} is the second discontinuity and, finally, t_{b0} is the time at which the force is back to zero amplitude. Notice that, t_{d1}^- and t_{d1}^+ are coincident for a slippage rupture, t_{d2} is only defined for tubes, and t_{d1}^+ coincides with t_{b0} for adhesions. All these points, excepted those lying in the baseline, are detected finding the extremes of the second derivative of the time-force curve, using the MATLAB function `findpeaks` (Fig. S11A).

Optical artefact correction

A typical force-curve should have a zero amplitude until the contact ($t = t_{c1}$). However, some curves come with a positive amplitude for $t < t_{c1}$ ($\sim$ 18% of the dataset), which has been understood as the signature of an optical artefact when part of the laser goes through the small T cell (see Fig. S11B, noised blue line between the two pink points). The detection of the artefact is based on a tolerance criterion of the max force-amplitude of this curve's segment, fixed to the mean noise force amplitude ($f_{oa}^{Tol} = 3$pN). The correction is based on the assumption of the symetry around $t = 0$ when the push and pull velocities are equal, the contact force is moderate and the contact time is small. Once detected, the artefacts were smoothed, mirrored, shifted at t=0 (blue smoothed line), and subtracted to the original force-curve (cyan), which gives the final corrected signal (orange).

Geometrical parameters

After the optical artefact correction, if any (see above), several geometrical parameters are then measured on each part of the curve (see Fig. S11A) and include contact/retraction slopes , force relaxation ($t_{c2} \leq t \leq t_{r1}$), slope of the linear part (around $t = 0$), slope of the tube ($t_{d1}^+ \leq t \leq t_{d2}$), force-drop between t_{d1}^- and t_{d1}^+, etc. . While the $t < 0$ part of the curve (in cyan, in Fig. S11) served for obtaining preliminary measures used as fitting guesses, the $t > 0$ part (in orange,) was exploited for fitting the model in Eq.2.

Discontinuity classification

Two types of discontinuities are identified : "rupture" and "slippage".

A rupture discontinuity is defined by the boolean defined by two logical conditions, on the absolute and relative value of the force drop at $t = t_{d1}$ (if any):

$$\texttt{ruptureON = FdropAbsNotWeakON \& FdropRelNotWeakON};$$

with the symbol `&` is the logical AND, and where

$$\texttt{FdropAbsNotWeakON} = |f_E(t_{d1}^-) - f_E(t_{d1}^+)| \geq f_0^{Tol};$$

$$\texttt{FdropRelNotWeakON} = f_E(t_{d1}^-)/f_E(t_{d1}^+) \geq 1 + R_f^{Tol};$$

$f_0^{Tol} = 3$pN corresponds to the average peak-to-peak amplitude of the experimentally recorded noise on the optical tweezer data $f_E(t)$. We fixed $R_f^{Tol} = 0.2$.

Slippage discontinuities are simply defined by the boolean

$$\texttt{slippageON} = \sim \texttt{ruptureON}$$

where $\sim$ is the logical negation.

Curve classification

Based on geometrical parameters measured on curves and on the characteristic points, four main categories of force curves are established: contact, adhesion, finite tube, infinite tube. This classification is performed with the requirement of several logical conditions, which are all referred to the positive-time domain of the force curve. First, a curve is classified as a contact (ie. not showing any significant event upon separating the cell and the bead) if the maximum or the mean force over $t > 0$ are found smaller with respect to a multiple of the force tolerance f_0^{Tol}. The logical condition is then

```
contactON = (FmaxWeakON || FmeanWeakON);
```

where the symbol `||` is the logical OR, and the two booleans are defined by:

$$\texttt{FmaxWeakON} = \max\{f(\forall t \leq t_{last})\} \leq 2f_0^{Tol};$$

$$\texttt{FmeanWeakON} = \text{mean}\{f(\forall t \leq t_{last})\} \leq f_0^{Tol}/2;$$

with $t_{last} = \min\{t_{b0}, t_{end}\}$.

Second, a curve is classified as an adhesion if the force amplitude drops to zero after the first event ($t = t_{d1}^-$), where the tolerance is now fixed on time, and corresponds to the minimum lifetime tolerance for tubes fixed to $\mathscr{T}_{tube}^{Tol} = 0.4$s (equivalent to a maximum length of 1µm). The logical condition, with the respective tolerances, is

```
adhON = ~ contactON & (F0aftert1ON & FbackTo0FastON);
```

where

$$\texttt{F0aftert1ON} = |t_{last} - t_{d1}^-| \leq \mathscr{T}_{tube}^{Tol};$$

$$\texttt{FbackTo0FastON} = t_{last} \leq 3\mathscr{T}_{tube}^{Tol};$$

This implicatess that, even if a curve shows a tube-like fingerprint, it will be classified as an adhesion if its lifetime is too short. The reason behind this choice is a matter of robustness : very short tubes are not very informative for performing a robust extrapolation of the tube parameters (k_{1N}, η_N), while they contains the information of the first elastic-like part of the model.

Finally, if none of the two previous conditions are trues, the curve is classified as a tube. The logical condition is then :

```
tubeON =  ~ (contactON || adhON).
```

Tubes are subsequently classified as “finite” or “infinite”, where “infinite” tubes are essentially those lasting until the end of the experiment (until $t = t_{end}$). In order to distinguish a tube from a residual weak force amplitude ($f_E \leq f_0^{Tol}$), the mean force of the tube and his final force are verified to be bigger than the previous zero-force tolerance. The logical definition is the following

```
infTubeON = tubeON & (FtubeNotWeakON & FendNotWeakON);
```

where

$$\texttt{FtubeNotWeakON} = [\mathrm{mean}\{f_{tube}\} \geq f_0^{Tol}/2]\,\&\,[f_E(t_{d2}) \geq f_0^{Tol}];$$

$$\texttt{FendNotWeakON} = f_E(t_{end}) \geq f_0^{Tol}/2;$$

and

$$f_{tube} = f_E(t_{d1}^+ \leq t \leq t_{d2}). \tag{8}$$

Finally, finite tubes are simply defined as

$$\texttt{finTubeON = } \sim \texttt{infTubeON}.$$

Curve fitting

Parameter estimation

Several geometrical parameters were used for determining preliminary estimations of the mechanical parameters. First, we measured directly from the experimental force-curve $f_E(t)$, the slope at contact $\frac{df_E}{dt}(t_{c1}) = \dot{f}_E(t_{c1})$ and at (negative force) retraction $\dot{f}_E(t_{r1})$, see Fig. S11, Inset, straight red and black lines, respectively. From these two slopes, we can obtain two estimations of k_2 (which are similar, with typical differences due to small hysteresis in retraction (5)). For this, we assumed that the deformation of the molecule is negligible for both contact and negative retraction situations ($k_1 \sim 0\,, \forall t < 0$). This is due to the fact that, differently from the pulling situation, pushing a single molecule from its equilibrium position leads to a negligible entropic contribution due to the molecular stiffness with respect to the pulling case.

Setting this condition in the model, and measuring the experimental force-slope of the negative retraction $\dot{f}_E(t_{r1})$, we get the following estimation of the cellular stiffness k_2

$$k_2^{est} \simeq \frac{\dot{f}_E(t_{r1})k_T}{k_T v_r - \dot{f}_E(t_{r1})}, \tag{9}$$

where we recall that v_r is the retraction velocity of the piezo-electric stage, and k_T is the stiffness of the optical trap. Obviously, only positive estimations where considered.

The same rationale conducted to the estimation of the whole elastic contribution of the system $k_{tot}^{est} = k_1^{est} + k_2^{est}$, from the experimental force-slope of the positive retraction $\dot{f}_E(t_{r2})$ (Fig. S11, inset, straight blue line). In fact, for the positive retraction case, we assumed that also the molecule is loaded together with the membrane, contributing to the total elastic stiffness, which is then estimated as

$$k_{tot}^{est} \simeq \dot{f}_E(t_{r2})\frac{k_T}{k_T v_r - \dot{f}_E(t_{r2})}.$$

As a consequence, one can obtain an estimation of the receptor/cytoskeleton bond stiffness from

$$k_1^{est} \simeq k_{tot}^{est} - k_2^{est}. \tag{10}$$

Moreover, the estimation of k_2 allows to estimate η in the waiting segment ($t_{c2} \leq t \leq t_{r1}$), by means of the direct fitting of the model with $k_1 \sim 0$ since the system is not under traction (Fig.S11, Inset, orange curve). An approximation of η is then given by

$$\eta^{est} = \frac{k_2^{est}k_T}{k_2^{est} + k_T}\frac{t_{r1} - t_{c2}}{\log[f_E(t_{c2})/f_E(t_{r1})]}. \tag{11}$$

Important enough, as the total effective contact time $t_C = t_{r1} - t_{c2}$ is not the same for all the curves, we chose to estimate η^{est} at $t_C = 0.4$ sec for the entire the dataset.

From Eq.4, with the limit $t \to +\infty$, we can find the approximation of the time-force curve for long tubes ($t_{d2} \gg t_{d1}^{+}$). This gives the approximation of the experimental tube slope at his end, $\dot{f}_E(t_{d2})$, from which we get the estimation of the tube stiffness as

$$k_{1N}^{est} \simeq \frac{k_T \dot{f}_E(t_{d2})}{k_T v_r - \dot{f}_E(t_{d2})}. \tag{12}$$

Finally, for long tubes the relaxation term of Eq.7, $e^{-\frac{k_2 \gamma_{1N}}{\eta_N}(t_{d2}-t_{d1})} \to 0$, which lead to the approximation of the force value at end of the tube, ie. at $t = t_{d2}$

$$f_E(t_{d2}) \simeq k_T v_r \frac{[\eta_N k_T + k_{1N}^{est}(k_{1N}^{est} + k_T)t_{d2}]}{(k_{1N}^{est} + k_T)^2}, \tag{13}$$

Considering $(k_{1N}^{est})^2 \ll k_{1N}^{est} < k_T$, we get

$$\eta_N^{est} \simeq f_E(t_{d2}) \frac{2k_{1N}^{est} + k_T}{v_r k_T} - k_{1N}^{est} t_{d2}. \tag{14}$$

Guessing values

Overall, the guess values for the mechanical parameters p^{guess} are fixed according to the estimated parameters p^{est} if any, or to a prefixed value otherwise. In the latter case, the prefixed values have been arbitrary fixed to the median of the estimated parameters $\mu(p_m^{est})$, calculated over all the m curves for which p^{est} exists. This case concerns only η_1 and k_{1N}, for which we have $\mu(\eta_1^{est}) \simeq 0.04$ pN nm^{-1} s, and $\mu(k_{1N}^{est}) \simeq 0.001$ pN nm^{-1}. Accordingly, the guessing values are generally fixed to

$$p^{guess} = \begin{cases} p^{est} & \text{if } \exists p^{est} > 0 \\ \mu(p_m^{est}) & \text{otherwise.} \end{cases} \tag{15}$$

Note that this rule is slightly modified for curves exhibiting tubes for the two time-dependent parameters k_1 and η, for which the rule becomes

$$p_1^{guess} = \max\{p_1^{guess}, p_{1N}^{est}\}.$$

This condition guarantees that $k_1^{guess} \geq k_{1N}^{guess}$ and $\eta^{guess} \geq \eta_{1N}^{guess}$, coherently with the fact that both stiffness and viscosity should not increase after the emergence of a tube These choices for the guessing values, even if not mandatory, increase the likelihood of a successful fit and consequently reduce the computational time.

Last, the guess value for the discontinuity event time is fixed to $t_{d1}^{guess} = t_{d1}^{-}$.

Curve fitting

The fitting was performed by means of the MATLAB function `fmincon`, which find the minimum of a constrained nonlinear function. This function was used for minimizing the residual sum of squares (RSS) between theoretical and measured forces. Accordingly, the objective function has been defined as

$$\mathscr{F} = \sum_{i=1}^{M} [f(t_r) - f_E(t_r)]^2, \tag{16}$$

where $\mathscr{F}$ is homogeneous to a force, and M is the total number of points constituting the fitted force curve.

The minimization procedure has been subjected to various constraints, defined as linear or nonlinear combination of the free parameters t_{d1}, k_1, k_2, η, k_{1N}, η_N. In particular, the constraints have been

imposed on (i) both the slopes of the time-force curve at the origin and at the end of the tube, and (ii) the force amplitudes at time t_{d1}^-, t_{d1}^+, t_{d2} as follows.

First, the slope of the force at the origin of times, defined as $\frac{df}{dt}(0) = \dot{f}(0) = \gamma_1 k_T v_r$, has been constrained to not differ by more than 10% from the experimental value of the slope at positive retraction $\dot{f}_E(t_{r2})$, hence:

$$0.9\,\dot{f}_E(t_{r2}) \leq \dot{f}(0) \leq 1.1\,\dot{f}_E(t_{r2}).$$

The constraint on the slope of the tube was

$$0.98\,\dot{f}_{tube} \leq \dot{f}(t \in (t_{d1}, t_{d2})) \leq 1.02\,\dot{f}_{tube},$$

where f_{tube} is defined in Eq.8. Second, the constraints on the forces at the first discontinuity have been fixed to

$$f_E(t_{d1}^-) \leq f(t_{d1}^-) \leq 1.2\,f_E(t_{d1}^-),$$
$$0.98\,f_E(t_{d1}^+) \leq f(t_{d1}^+) \leq 1.02\,f_E(t_{d1}^+),$$

and at the second discontinuity was

$$0.98\,f_E(t_{d2}^-) \leq f(t_{d2}^-) \leq 1.02\,f_E(t_{d2}^-).$$

For the maximum force peak before transition, $f_E(t_{d1}^-)$, we fixed a bigger tolerance with respect to the other values because a small subset of curves present a fast change on the force-slope preceding the discontinuity at $t = t_{d1}^-$. This change does not correspond to the relaxation term introduced by the viscous dashpot, and - for preserving simplicity - we choose to not account for this (occasional) behaviour.

Finally, the discontinuity in $t = t_{d1}^-$ has been modeled as a "degradation" of both the molecular elastic (k_1) and cellular viscous (η) parameters, for which we imposed that $k_{1N} \leq k_1$ and $\eta_N \leq \eta$.

To avoid potential non-physical solutions, we defined a set of lower and upper bounds (lb, ub) for all the parameters, so that the fitting solution of a parameter p is always in the range $p^{lb} \leq p \leq p^{ub}$. For the majority of the parameters, we fixed the lower/upper bounds to very small/big values (see Table 2) with respect to their final median (see Table 1, main text).

Table 2: Upper and lower bounds for each fitting parameter

bounds	t_1	k_1	k_2	η	k_{1N}	η_N
lower	$0.95\,t_{d1}^-$	10^{-5}	10^{-5}	$0.1\,\mu(\eta_1^{est})$	10^{-5}	10^{-5}
upper	$1.05\,t_{d1}^-$	10^2	10^2	$10\,\mu(\eta_1^{est})$	10	10

For the particular choice of t_1 and η bounds, we did as follow. First, we limited t_{d1} to a very narrow region around the point spotted on the curve $(0.95\,t_{d1}^-,\ 1.05\,t_{d1}^-)$, the transition being generally well identified for the majority of force curves. Second, we limited η to the region around the median of its estimated value $(\frac{1}{10}\,\mu(p_m^{est}), 10\,\mu(p_m^{est}))$, due to the large variance of the corresponding distribution. This has two counterparts : from the one hand, it makes risky to fix η exactely to its median; on the other hand, too small or too big values of η can lead to a failure of the fitting algorithm. The great variance of η^{est} reflects the difficult to extrapolate this parameter, which is mostly related to the quasi-linear behaviour of the majority of the force-curves where the term $e^{-\frac{k_2\gamma}{\eta}t}$ approaches zero.

Fit quality and residuals

The difference between the experimental and the fitted curve has been evaluated in term of the residual standard error (RSE), obtained by taking the square root of the objective function of Eq.16 normalized by $M-2$. The RSE was separately evaluated before and after the first discontinuity (corresponding to $t = t_{d1}^-$) such as

$$\mathscr{F}_{A,B}^{Tol} = \sqrt{\frac{1}{M_{A,B}-2}\sum_{i=1}^{M_{A,B}}\left[f_{A,B}(t_r) - f_E(t_r)\right]^2}, \tag{17}$$

where, $f_A(t)$ and $f_B(t)$ correspond to Eq.6 and Eq.7, respectively. Accordingly, M_A and M_B represent the number of points of the force curve for $0 \leq t \leq t_{d1}^-$ and $t_{d1}^- < t \leq t_{d2}^-$.

Based on the RSE values, we evaluated the fit quality of each curve with the following boolean

`BigResON = ( BigResON-A || (tubeON & BigResON-B) ),`

where

`BigResON-A,B =` $\mathscr{F}_{A,B} \geq \mathscr{F}_{A,B}^{Tol}$,

with $\mathscr{F}_A^{Tol} = 6$ pN $= 2 \times f_0^{Tol}$, $\mathscr{F}_B^{Tol} = \frac{1}{2}\mathscr{F}_A^{Tol} = f_0^{Tol}$.

Data post processing

The post processing of the data is based on the following python librairies, mainly avalaible in Anaconda Python Distribution (https://anaconda.org/ ; numpy, scipy, scikit, pandas) or on the web (dabest ; ttps://acclab.github.io/DABEST-python-docs/index.html (4)). The visualisation has been made with matplotlib, seaborn (https://seaborn.pydata.org/index.html) and dabest packages.

Data preparation, sorting and cleaning

We first loaded the data output by the fitting and classification procedure as .xls, from the different experimental sets, and curated it for easy further processing. We then used booleans present in the data file to remove curves having been labelled by the entire procedure as rejected (eg. because of too large fitting residuals).

Comparing at that stage the differents data sets (slightly different k_T, contact forces 10-15pN) we observed that in the experimental ranges, neither the dispersion of the fitted parameters nor the central tendancies depend on the initial data setdata. We then confidently pooled all data sets in further analysis.

We then subsetted the data to short contact times, between 0 and 0.5 sec, to be sure to have mainly unique tubes in our analysis.

From the fitting strategy we presented in the relevant section, the following pooling of data have been made [see Table 1 in the main text]. The fit has been faithfully estimating k_2 for rupture and slippage tubes ; k_1 for rupture tubes only, k_{1N} for rupture and slippage tubes ; eta for rupture and slippage tubes ; etaN for rupture and slippage tubes. This allowed us to plot, separating each antibody case with or without latrunculin, the final population of acceptable values. We present the obtained data sets in the Fig. 3 in the main text and in the SI, in particular Fig. S6.

Data representation and statistical tests

We chose to use a Data Analysis with Bootstrap-coupled ESTimation strategy (`dabest` Python package) (4).

We set to evaluate (a) the relative variations of the parameters estimated without latrunculine among the different antibodies used as handles to pull adhesion events or tubes to detect the molecule effect on the different mechanical parameters, and (b) the relative variation, for each parameter and antibody, of the value with vs. without the drug presence as an indicator of the cytoskeleton on each parameter, for each molecule.

This methodology allows to represent the dataset explicitely and uses a bootstraping approach to estimate the distribution of the differences between two sets of data (eg. between without and with

latrunculin for a given parameter and a given molecules) or between one reference and other data sets (eg. Comparing aCD3 to each of the others antibodies).

The estimation plot produced allows to conclude if, for a given CI value (here 95%), data sets are extracts of different or not populations. Where a data set was observed to be significantly different (in terms of dabest analysis) from its comparison / reference distribution, we indicated it on the graphs by a star symbol (*).

Pooling Adhesion and tube data

Mechanical parameters can be obtained in principle from the adhesion curves ()see Table 1 in the main text), but with a reduced accuracy, in particular for k_1. This is illustrated on Fig. S3, where the dispersion can be appreciated. This dispersion implicates that some of the significant differences observed for the tubes only data are affected, but not the relative variations of their median values. As a consequence, we did not pool the adhesion data with the Rupture case.

SI References

(1) Tolić-Nørrelykke SF, et al. (2006) Calibration of optical tweezers with positional detection in the back focal plane. Review of Scientific Instruments 77(10):103101.

(2) Lim C, Zhou E, Quek S (2006) Mechanical models for living cells—a review. Journal of Biomechanics 39(2):195–216.

(3) Schmitz J, Benoit M, Gottschalk KE (2008) The Viscoelasticity of Membrane Tethers and Its Importance for Cell Adhesion. Biophysical Journal 95(3):1448–1459

(4) Ho J, Tumkaya T, Aryal S, Choi H, Claridge-Chang A (2019) Moving beyond P values: Data analysis with estimation graphics. Nature Methods 16(7):565–566.

(5) Nawaz S. et al (2012) Cell Visco-Elasticity Measured with AFM and Optical Trapping at Sub-Micrometer Deformations. PLoS ONE 7(9):e45297.

(6) Limozin L, Puech PH (2019) Membrane Organization and Physical Regulation of Lymphocyte Antigen Receptors: A Biophysicist's Perspective. The Journal of Membrane Biology 252(4- 5):397–412.

Supplementary Figures

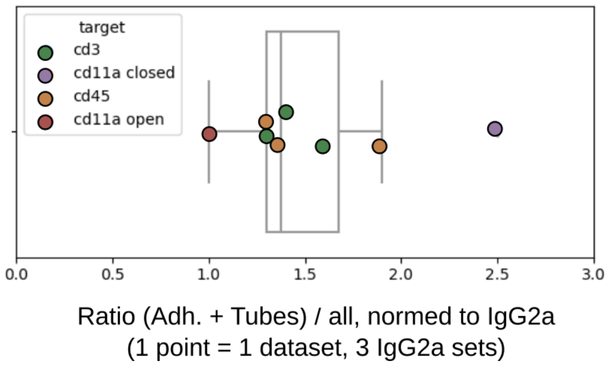

Figure S1: Specificity of the different antibody handles, compared to IgG2a isotype control. The graph presents the ratio of (adhesion+tubes) to the total number of curves, per handle molecule. Note that since our Jurkat T cells are not activated, the number of interactions that was recorded with the antibody directed toward the open conformation of LFA1 was low, and lower than for the closed state of this integrin (6).

.

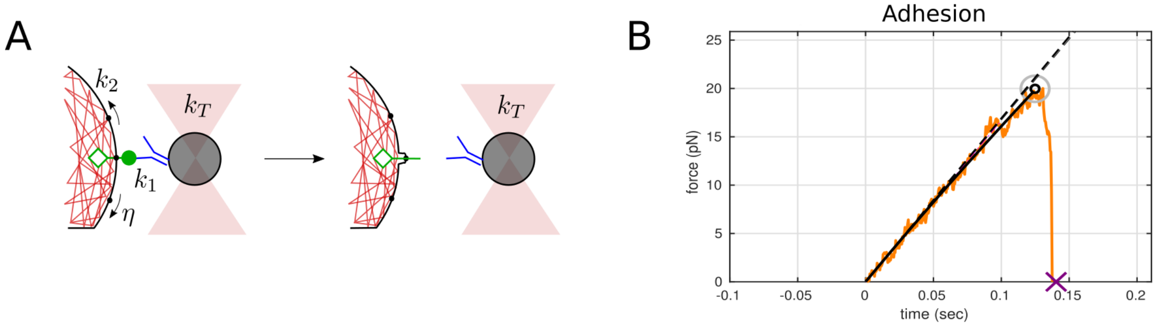

Figure S2: Microscopic interpretation of the force curve for the Adhesion case. (**A**) The rupture of the receptor-antibody link occurs before the tube formation. (**B**) Corresponding 'adhesion' force vs time curve showing a single slope before the force goes back abruptly to the baseline.

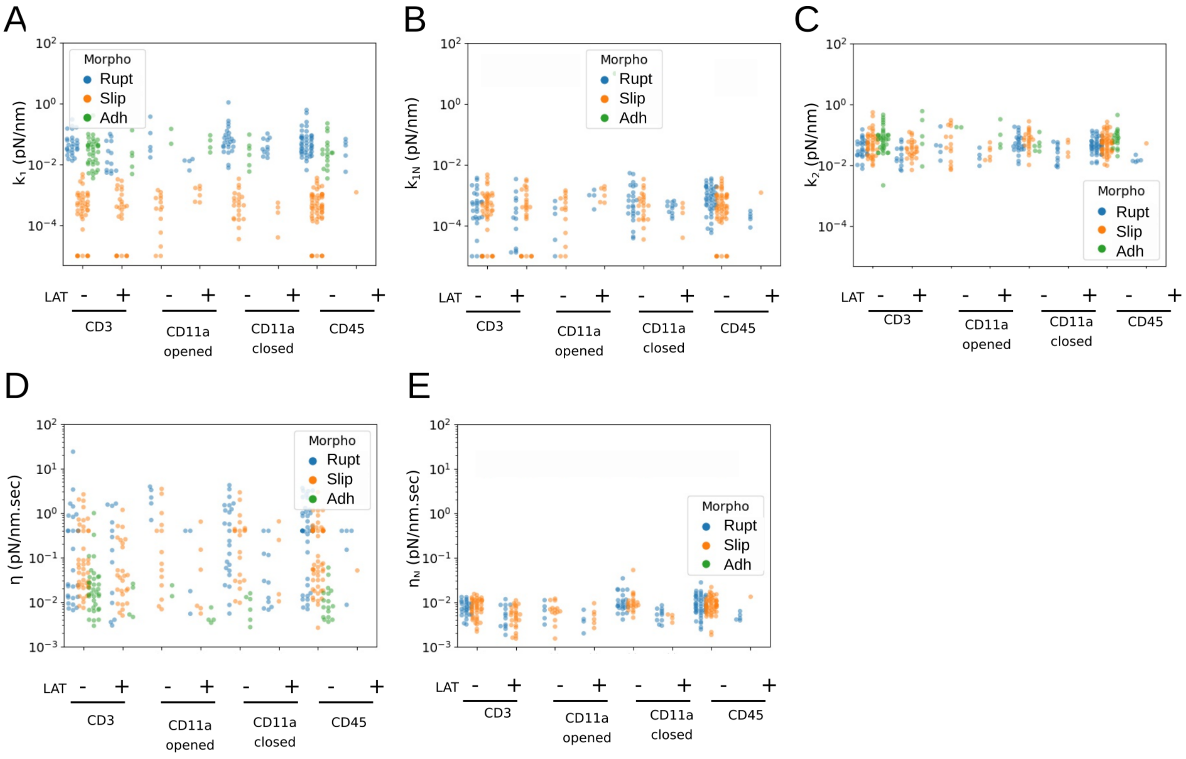

Figure S3: Scatter plots of all mechanical parameters, extracted from the experimental data, as a function of the antibody handle, presence or not of LatA treatment and morphology (Rupt = ”rupture” tube, Slip = ”slippage” tube, Adh = ”adhesion”). Please note that the k_{1N} and η_N values for adhesion curves are not existing by model definition. One point corresponds to one fitted curve.

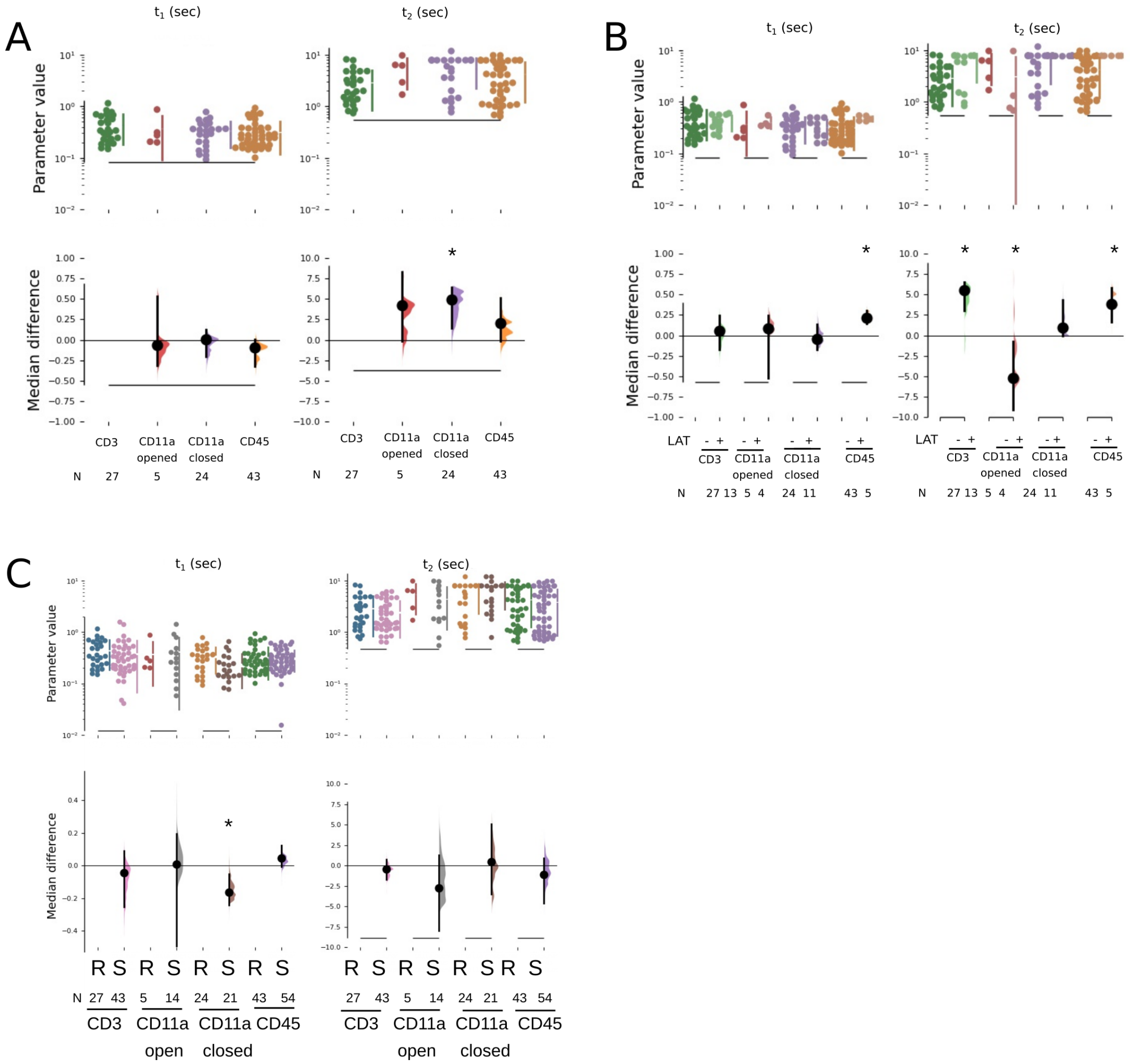

Figure S4: (**A**) Measurements of parameters and median difference estimation plots for t_1 and t_2, taking CD3 as a reference, for "rupture" tubes only. (**B**) Measurements and estimation plots for the same parameters, including the data where latrunculine was used. Here, the comparison is made between the cases without and with the drug, for each antibody handle. (**C**) Comparison, per antibody handle, between "rupture" (R) and "slippage" (S) tubes. One point corresponds to one fitted curve. N is the number of curves per condition.

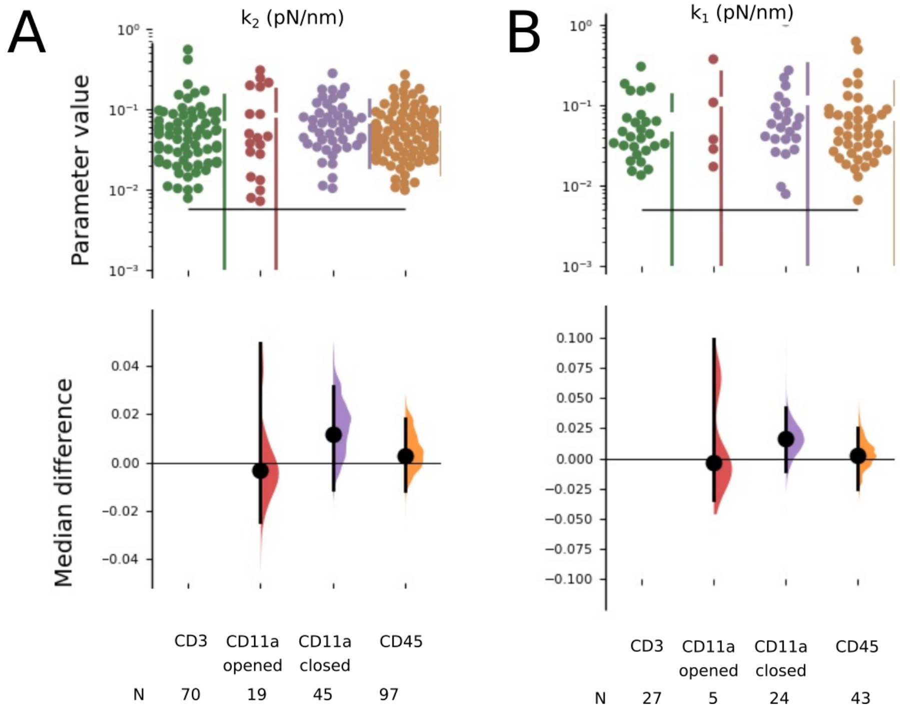

Figure S5: Measurements of parameters and estimation plots for k_2 (**A**) and k_1 (**B**), for each targeted membrane receptor without Latrunculin treatment. The median difference is calculated relatively to the condition targeting CD3, serving as a reference. One point corresponds to one fitted curve. N is the number of curves per condition.

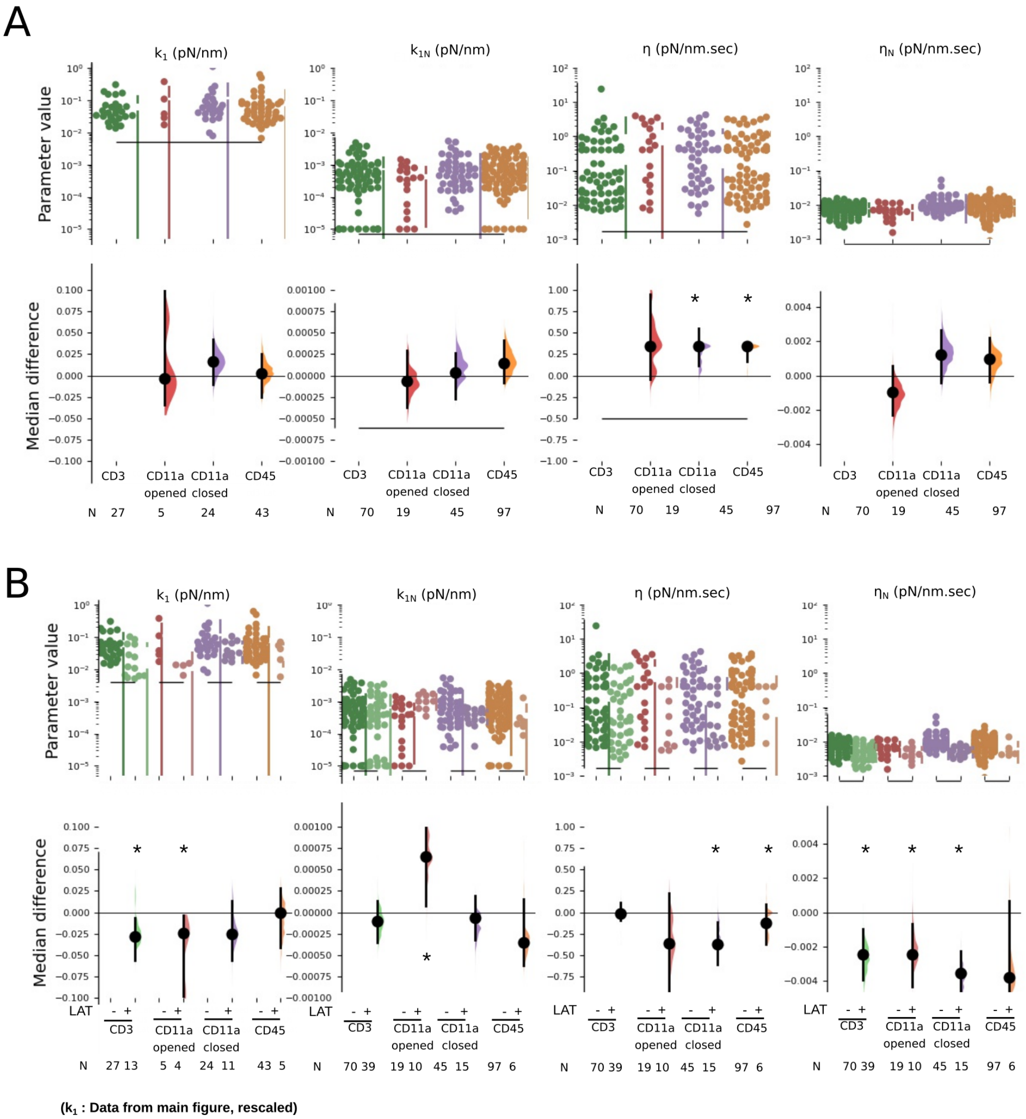

Figure S6: Measurements of parameters and estimation plots for k_1 (reproduced from main text data), k_{1N}, η and η_N and for each targeted receptor. (**A**) Without LatA treatment, relatively to CD3 as a reference. (**B**) to compare, for each antibody, the cases +/- Latrunculin. One point corresponds to one fitted curve. N is the number of curves per condition.

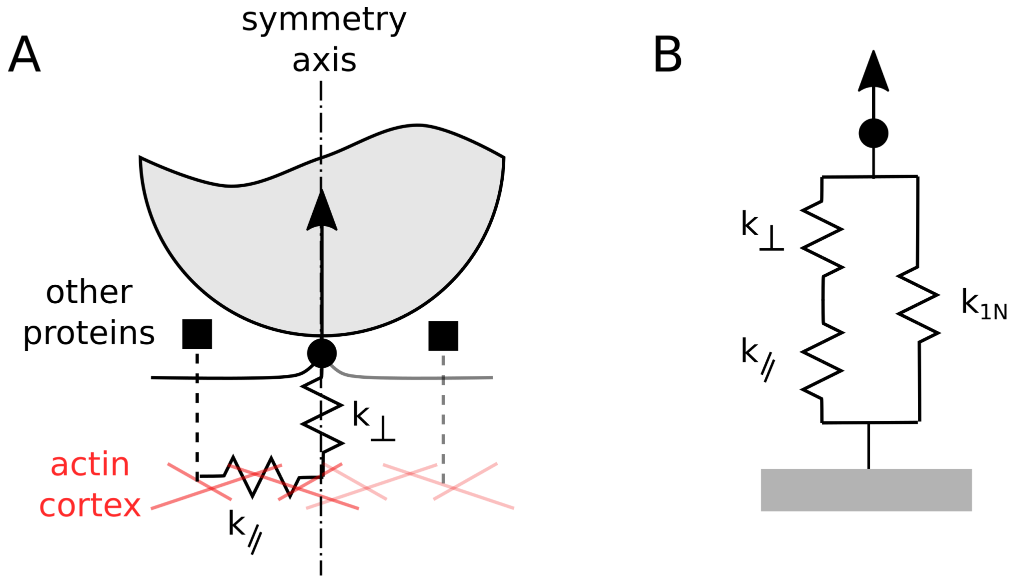

Figure S7: A : Schematics of the early moments of tube pulling. B : Equivalent spring-based model
.

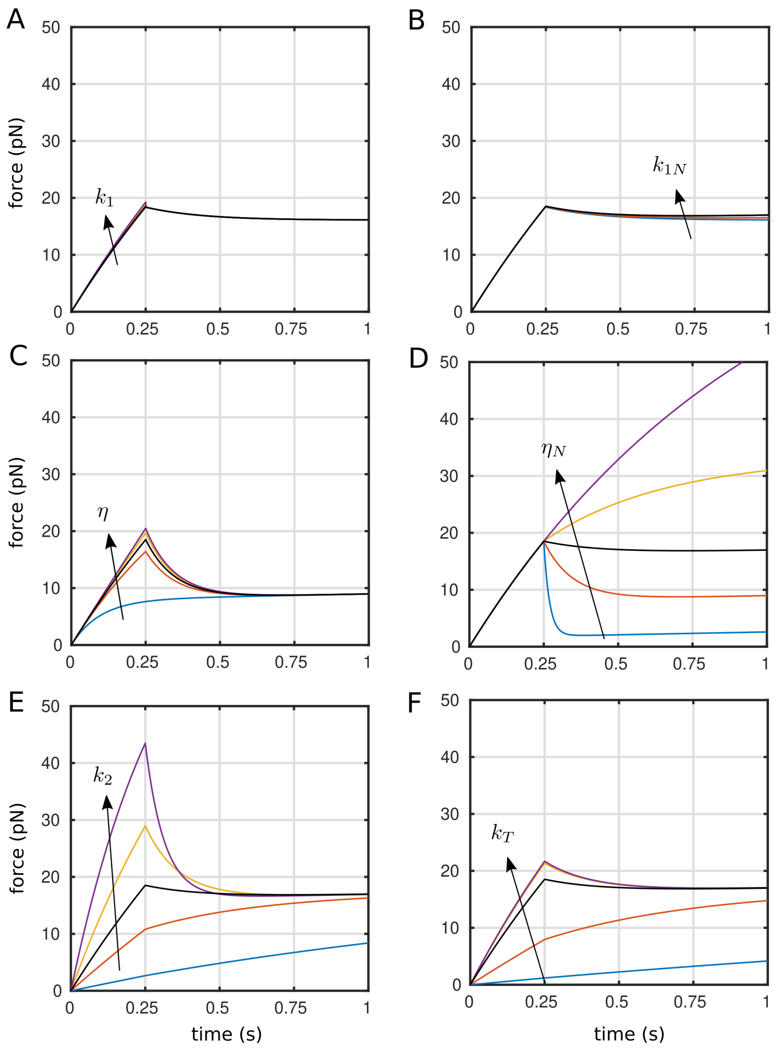

Figure S8: Parametric study of the viscoelastic model for the slippage case. Reference model parameters are obtained from the fit of the experimental 'slippage' curve in Fig. 2D: $v_r = 2000\text{nm}\,\text{s}^{-1}$, $k_T = 0.25\text{pN}\,\text{nm}^{-1}$, $k_1 = k_{1N} = 0.0005\text{pN}\,\text{nm}^{-1}$, $\eta_N = 0.008\text{pN}\,\text{nm}^{-1}\,\text{s}$, $k_2 = 0.05\text{pN}\,\text{nm}^{-1}$, $\eta = 0.04\text{pN}\,\text{nm}^{-1}\,\text{s}$, , and $t_1 = 0.25\text{s}$. For panels **A**) to **E**), the corresponding reference curve is shown in black while the other curves have been obtained by multiplying by 0.1, 0.5, 1, 2 and 5 the reference parameter value: (**A**) k_1; (**B**) k_{1N}; (**C**) η; (**D**) η_N; (**E**) k_2. For panel **F**), k_T = 0.01, 0.1, 1, 10 or 100 $\text{pN}\,\text{nm}^{-1}$ and the other parameters have their reference value.

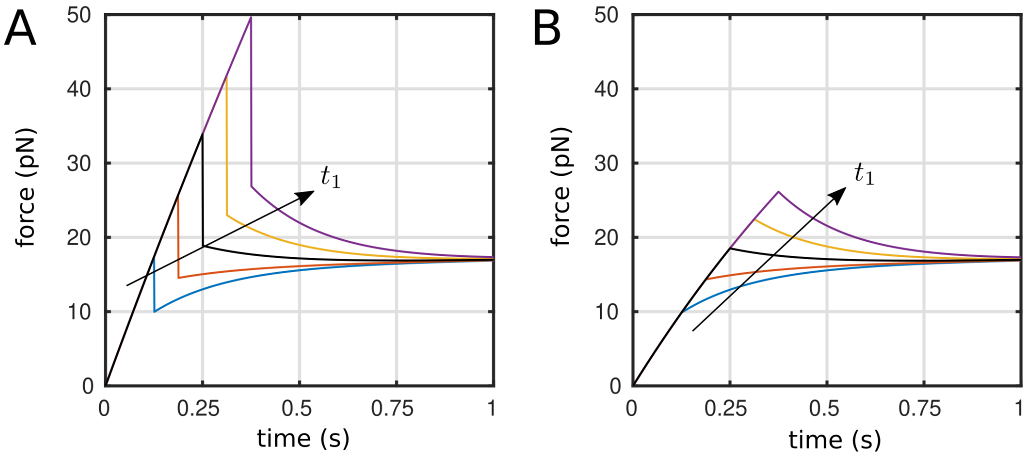

Figure S9: Parametric study of the viscoelastic model for the time of the discontinuity t_1. A: Rupture case. B: Slippage case. The black curve in between the others correspond to the one obtained via the fitting in Fig. 2, left panel. Parameters are $v_r = 2000\text{nm}\,\text{s}^{-1}$, $k_T = 0.25\text{pN}\,\text{nm}^{-1}$, $k_1 = 0.05\text{pN}\,\text{nm}^{-1}$, $k_2 = 0.05\text{pN}\,\text{nm}^{-1}$, $\eta = 0.04\text{pN}\,\text{nm}^{-1}\,\text{s}$, $k_{1N} = 0.0005\text{pN}\,\text{nm}^{-1}$, $\eta_N = 0.008\text{pN}\,\text{nm}^{-1}\,\text{s}$, and $t_1 = 0.25\text{s}$. The others curves have been obtained multipling the value of t_1 by the following vector of factors $\{0.1, 0.5, 1, 2, 5\}$.

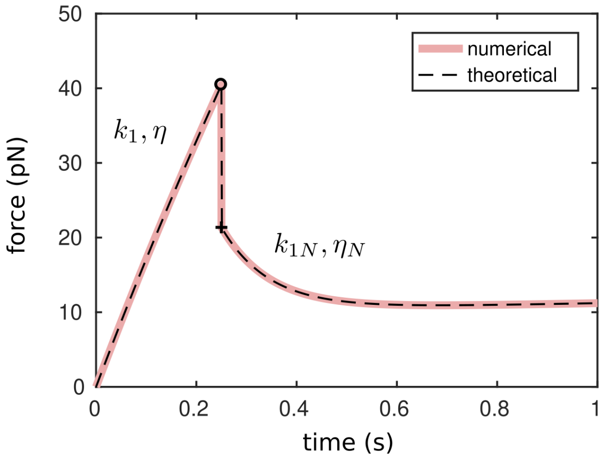

Figure S10: Theoretical force vs time curve for a membrane tube extraction experiment, corresponding to the rupture case. Numerical (red curve) and analytical **(dotted-black curve, Eq. SI 7)** results were obtained with $x_0 = 0\text{nm}$, $v_r = 2.5\mu\text{m}\,\text{s}^{-1}$, $t_r = 0$ sec, and $f_0 = 0$ pN and $t_d = 0.25$**s**. The force history is caracterized by two regimes : for $0 \leq t \leq t_d$ an almost linear regime followed by a very moderate relaxation, at $t = t_d$ an instantaneous release of the force, due to the abrupt change in stiffness $k_1 \to k_{1N}$, and - finally - for $t > t_d$ a second relaxation followed by a quasi-plateau of the curve.

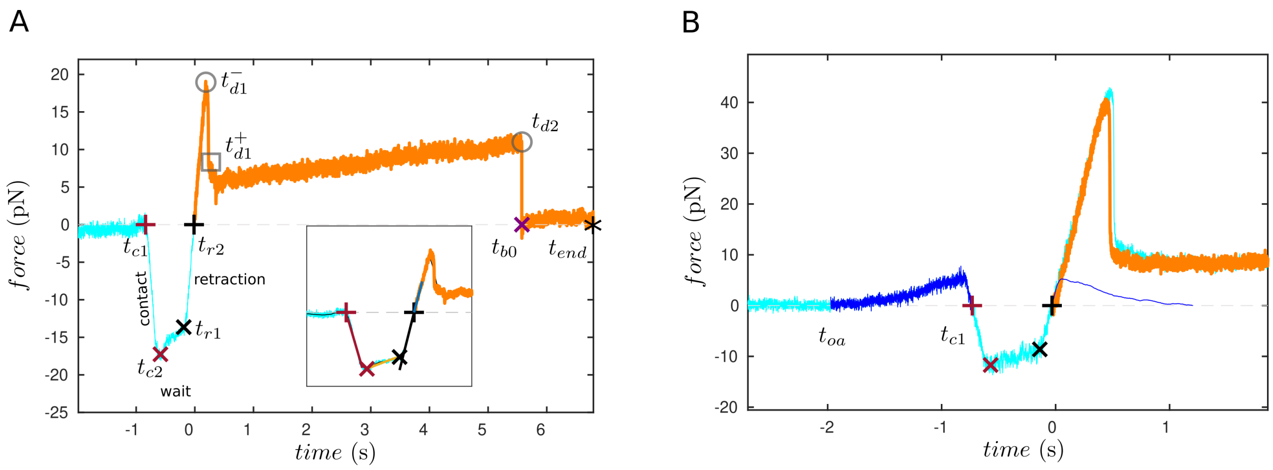

Figure S11: Experimental force-curve for a “rupture” event. (**A**) Characteristic points spotted on a typical curve. t_{c1} and t_{c2} are the time points at which the contact starts and ends respectively (in red); t_{r1} and t_{r2} are the start/end retraction points (in black); t_{d1}^- and t_{d1}^+ are the first discontinuity in the force curve during retraction (left and right limits), t_{d2} is the second discontinuity and, finally, t_{b0} is the time at which the force is back to zero amplitude. Notice that, t_{d1}^- and t_{d1}^+ are coincident for a slippage rupture, t_{d2} is only defined for tubes, and t_{d1}^+ coincides with t_{b0} for adhesion curves. (**B**) Correction of an optical artifact. Light blue: original data; dark blue: data between t_{oa} and t_{c1} optical artifact on the pressing segment of the curve, average and symetrized for the pulling on segment (blue thin line); orange: corrected data on pulling segment.
